# A neuro-synthesis of psychological constructs relevant to posttraumatic stress disorder

**DOI:** 10.1038/s41598-026-60710-8

**Published:** 2026-07-09

**Authors:** Resh S. Gupta, Alan N. Simmons, Daniel M. Stout

**Affiliations:** 1 Department of Psychology and Human Development, Vanderbilt University, Nashville, TN 37240 USA; 2 Center of Excellence for Stress and Mental Health, VA San Diego Healthcare System, San Diego, CA 92161 USA; 3 Department of Psychiatry, University of California San Diego, San Diego, CA 92093 USA

**Keywords:** PTSD, Neuroimaging, Network, Neurosynth, fMRI, Neuroscience, Psychology, Psychology

## Abstract

Post-traumatic stress disorder (PTSD) is a heterogeneous neuropsychiatric disorder defined by a constellation of symptoms and psychological processes following trauma exposure. PTSD research spans diverse constructs, but heterogeneous terminology and task-specific findings complicate efforts to link these constructs to neural systems. While neuroimaging meta-analyses have revealed structural, functional, and network abnormalities in PTSD, few studies have examined how PTSD-relevant psychological constructs are organized according to broader meta-analytic activation patterns. To address this gap, we combined Neurosynth and PubMed text mining to (1) identify psychological construct terms that are represented in PTSD research discourse and have sufficient neuroimaging meta-analytic support, and (2) determine whether these constructs cluster into neuroimaging-informed domains that align with canonical brain networks. Using graph network and community detection clustering, we identified four stable clusters that were labeled “Cognition,” “Emotion,” “Memory Function,” and “Decision-Making.” The cognition cluster converged on middle frontal gryus, amygdala, and dorsal anterior insula. The emotion cluster mapped onto the amygdala, subgenual anterior cingulate (sgACC), and ventromedial prefrontal cortex (vmPFC). The memory function cluster involved hippocampus, amygdala, and vmPFC. The decision-making learning cluster encompassed vmPFC, striatum, and sgACC. By integrating Neurosynth term-based meta-analyses with PubMed filtering, network-based clustering, our approach bridges semantic, mechanistic, and neural organization, reducing conceptual fragmentation providing a neuroimaging informed conceptual framework of PTSD-related discourse.

**Supplementary Information:**

The online version contains supplementary material available at https://doi.org/10.1038/s41598-026-60710-8.

## Introduction

Post-traumatic stress disorder (PTSD), a debilitating neuropsychiatric consequence of exposure to traumatic events, is highly prevalent^1^ and is associated with increased morbidity and mortality^2^. Hallmark symptoms of PTSD include mnemonic and affective intrusions associated with the traumatic event, avoidance of trauma-related cues, alterations in cognition, mood, and arousal, and hyperarousal symptoms^3^. Although efficacious, empirically supported psychological treatments such as Prolonged Exposure (PE) and Cognitive Processing Therapy (CPT) are not universally well-tolerated^4^, and up to two-thirds of patients experience residual symptoms and still meet diagnostic criteria for PTSD after treatment^5^. Given the high personal and economic burden associated with PTSD, there is a critical need to understand the underlying neural mechanisms associated with its etiology, maintenance, and treatment.

PTSD is characterized by persistent alterations in multiple neurocognitive and affective mechanisms^6^. For example, individuals with PTSD frequently exhibit hypervigilance toward threat cues^7^, impaired extinction of conditioned threat^8,9^, deficits in attentional control^10^, and blunted responsiveness to positive or rewarding stimuli^11^. Neurobiologically, these symptom domains map onto several large-scale circuits: salience and attentional‐control networks (e.g., dorsal anterior cingulate cortex, anterior insula, dorsolateral prefrontal cortex^12,13^, limbic emotion‐regulation systems (e.g., ventromedial prefrontal cortex, amygdala^14^, hippocampal‐prefrontal memory circuits^15,16^, and mesolimbic reward pathways (e.g., ventral striatum, orbitofrontal cortex^17,18^.

Despite extensive task-based and resting‐state functional magnetic resonance imaging (fMRI) studies, PTSD findings remain fragmented. One major impediment is the widespread use of heterogeneous terminologies for constructs across studies; terms such as “attentional bias,” “threat vigilance,” “distractor interference,” and “conflict monitoring” may refer to overlapping cognitive processes but are often operationalized via distinct paradigms^19–21^. Similarly, constructs such as “contextual memory,” “extinction recall,” “autobiographical retrieval,” and “pattern separation” recruit substantially overlapping neural circuits^22–24^, yet are discussed under different theoretical umbrellas. Because researchers often use different terms for overlapping phenomena, it can be difficult to determine whether separate studies are uncovering unique psychological processes or just describing the same neural systems in different ways^25,26^. This inconsistency in the terms used to describe psychological constructs makes it challenging to compare results across studies and hinders progress toward a unified model of PTSD.

**Fig. 1:**
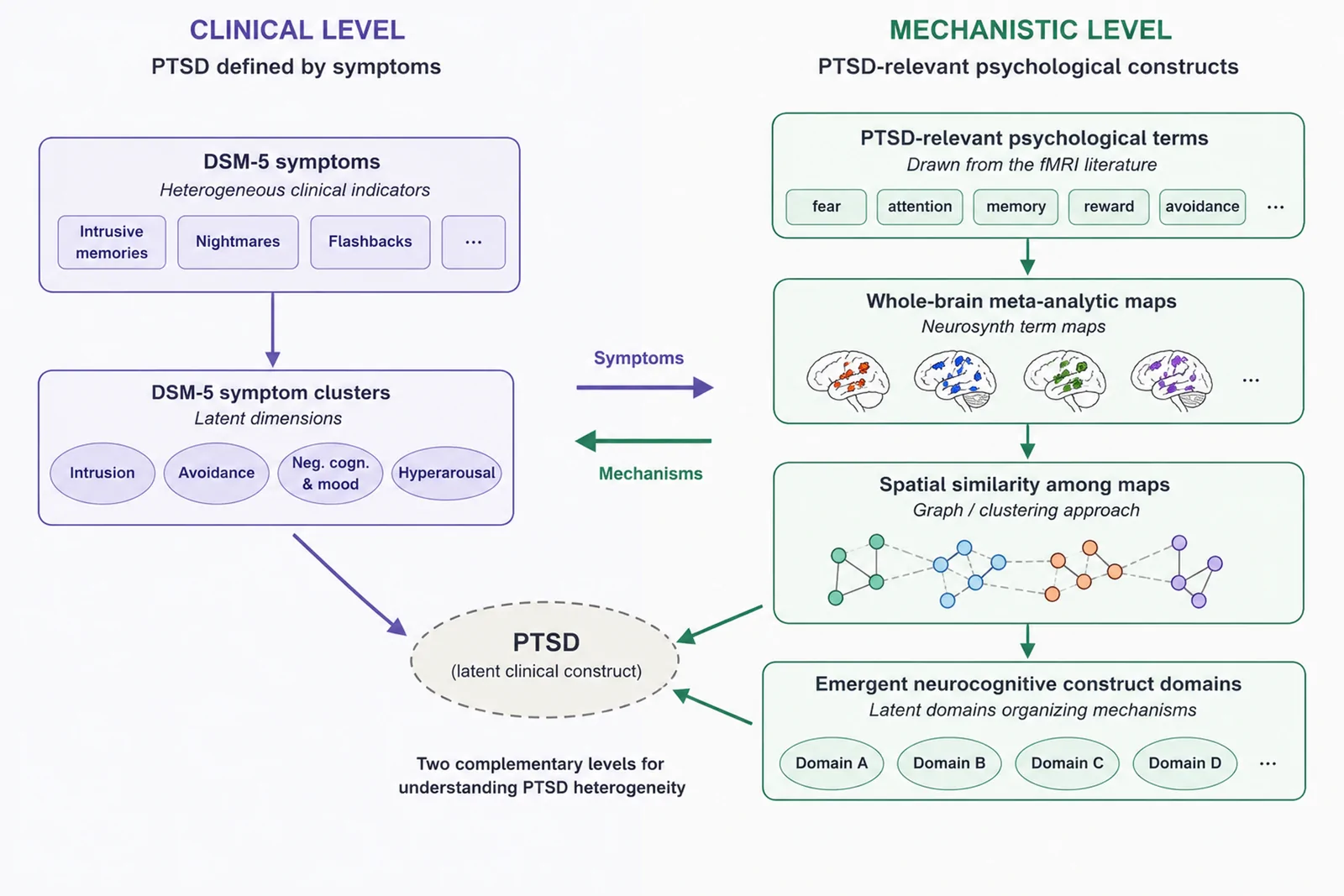
Conceptual framework. Similar to the formulation of PTSD in the DSM-5, the fMRI- based model starts with a selection of concepts, terms, and symptoms that are conceptually linked to PTSD. From this clusters are formulated that represent latent constructs within PTSD. These latent constructs are the foundation upon which the concept of PTSD is predicated. In this way the fMRI meta-analytic-based approach (right side) provides a similar approach to the DSM clinically based approach (left side).

PTSD is not a singular biological entity, but a diagnostic construct defined by the co-occurrence of heterogeneous symptoms, where the label “PTSD” is applied when a particular constellation of behaviors meets threshold criteria^27,28^. Rather than reflecting a single underlying dysfunction, these symptoms are thought to emerge from alterations in multiple neurocognitive processes^6^. This implies that PTSD does not have a single, unified neural representation; but instead reflects the combined influence of multiple interacting processes. As a result, attempts to map PTSD at the level of the disorder risk collapsing across these mechanisms, thereby obscuring the neural architecture that gives rise to its symptom profile. An alternative strategy is to reconstruct PTSD from its constituent mechanisms, treating PTSD as an emergent organization of core neurocognitive processes. This perspective shifts the focus from localizing PTSD as a unitary entity to characterizing the latent organization of the mechanisms that collectively define it (see Fig. 1).

Meta-analytic platforms such as Neurosynth offer an automated way to aggregate thousands of published fMRI studies into term‐based activation maps^29^. This approach differs from traditional coordinate-based meta-analyses, such as activation likelihood estimation, which typically use manually curated studies to test spatial convergence for a specific experimental contrast or diagnostic comparison^30^. In contrast, Neurosynth uses automated text mining to link psychological, anatomical, and methodological terms to reported activation coordinates across a much broader literature, making it particularly useful for examining how constructs are represented across neuroimaging studies. Each map reflects where brain activation has been most consistently reported in studies mentioning a given keyword (e.g., “amygdala,” “fear-conditioning,” “working memory”), with voxelwise Z-scores indexing the strength of association between the term and activation across the literature. However, the variability in how psychological constructs are defined and mapped onto neural activity obscures their underlying structure, necessitating data-driven approaches to identify coherent, higher-order psychological domains and their associated neural systems^31^. Such approaches would allow investigators to move beyond fragmented terminology, improving interpretability, facilitating cross‐study comparisons, and guiding more focused hypotheses and interventions^32^. For example, Alhazmi et al. (2018) applied hierarchical clustering to 3114 terms across Neurosynth and derived six primary semantic domains, each linked to characteristic patterns of brain activation, demonstrating the utility of organizing constructs based on shared neural signatures rather than surface-level terms^33^. Despite these advances, few studies have systematically applied such approaches to organize the diverse psychological constructs relevant to PTSD and link them to core neural substrates across the neuroimaging literature^34–36^. Traditional coordinate-based meta-analytic approaches (i.e., activation likelihood estimation) primarily localize convergent activation patterns but are less suited for organizing the psychological constructs that give rise to those patterns.

Our study aimed to characterize the latent organization of neurocognitive mechanisms implicated in PTSD by clustering meta-analytic brain maps of psychologically meaningful constructs based on shared neural signatures. To this end, we employed an analytical approach to overcome terminological heterogeneity and link meta-analytic neural concept templates to PTSD‐relevant psychological constructs (see Supplementary Fig. 1 for a flow diagram of analytical plan). We first identified Neurosynth terms corresponding to psychological constructs, excluding purely anatomical or directional labels (e.g., “amygdala,” “insula,” “left,” “right”), and filtered this set to constructs frequently represented in PTSD‐focused PubMed abstracts. This ensured that each included activation map reflected a construct of established relevance to PTSD discourse while preserving the brain map’s normative neural representation. We then applied hierarchical clustering to these construct-level maps to identify latent higher-order concepts that reflect shared neural organization across related psychological mechanisms. This approach leverages normative neural representations of psychological constructs to characterize the systems while simultaneously resolving semantic overlap across constructs that may reflect shared underlying mechanisms. This study addresses three important gaps: (1) it provides a principled framework resolving inconsistencies in construct terminology by organizing constructs according to shared neural architecture; (2) it identifies neurobiologically grounded domains that can guide region‐of‐interest (ROI) selection, hypothesis generation, and cross‐study integration^37^; and (3) it maintains clinical relevance by anchoring these domains in constructs frequently studied in PTSD, while leveraging transdiagnostic meta-analytic data ^29^.

## Results

**Fig. 2:**
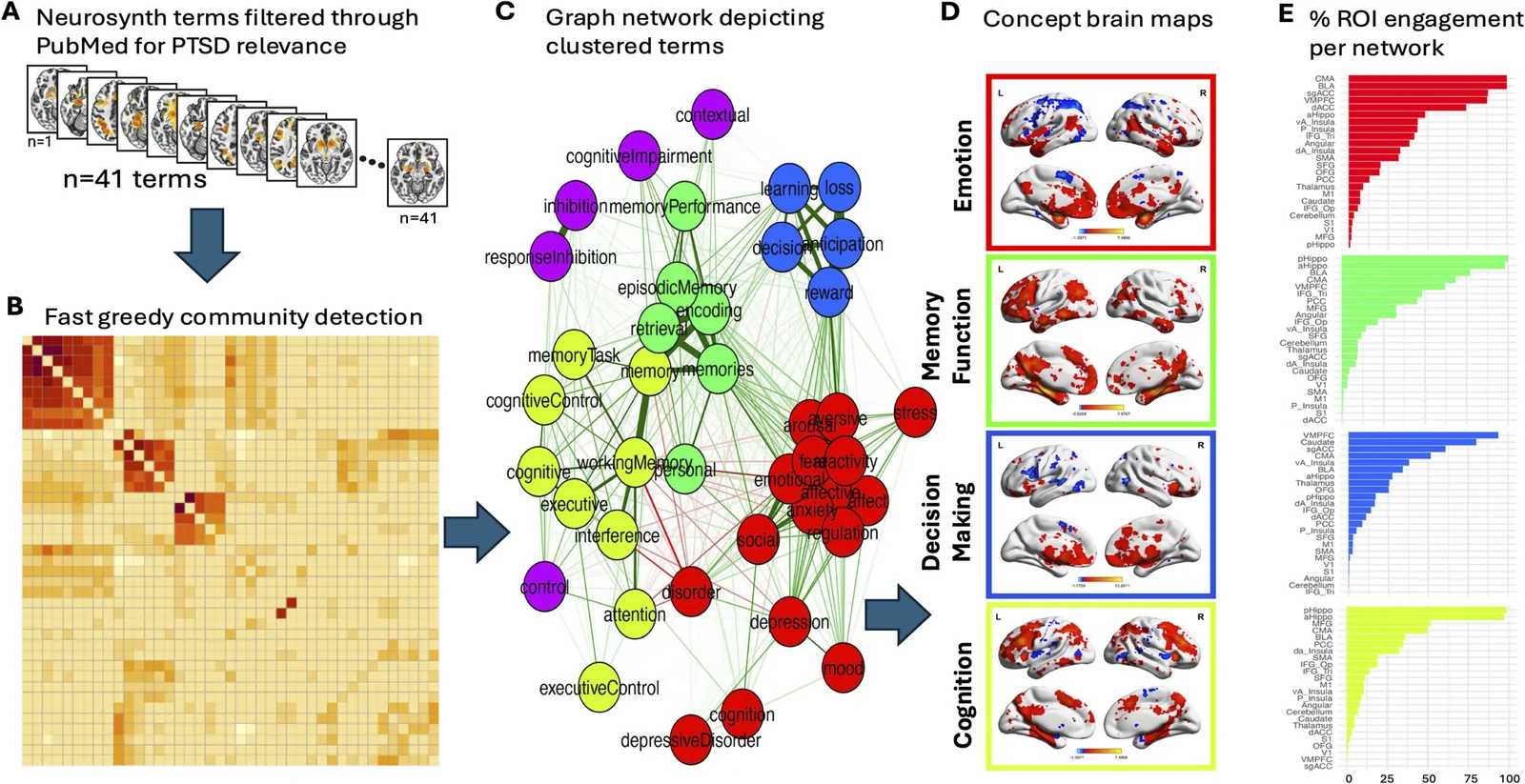
Analysis and results overview. (**A**) Clustering of the 41 terms, performed with a binary graph map (top 15%), yielded four meaningful conceptual clusters: cognition, emotion, memory functioning, and decision. making (**B** & **C**). Every term within the cluster was averaged to create brain maps for each conceptual cluster (**D**). The “independent” category captured terms that did not form their own cluster and were not strongly associated with the four meaningful clusters (**C**). Identification of % ROI engagement for each conceptual concept for interpretation (**E**). % ROI engagement was defined as the number of voxels out of the total voxel count for the listed ROIs that overlapped with the voxels from the respective conceptual brain map.

As Shown in Fig. 2A, clustering of the 41 terms, performed with a binary graph map (top 15%), yielded four meaningful clusters: cognition, emotion, memory functioning, and decision making (see Fig. 2B,C). To visualize these four clusters, we averaged the terms within each cluster to create brain maps for each cluster (see Fig. 2D). These concept brain maps provide a neurobiologically informed representation of psychological mechanisms that have been extensively investigated in PTSD. As shown in Fig. 2E, the emotion concept cluster has prominent activation in the centromedial (CMA) and basolateral amygdala (BLA), the subgenual anterior cingulate cortex (sgACC), dorsal anterior cingulate cortex (dACC), and the ventromedial prefrontal cortex (VMPFC). The memory functioning concept cluster shows prominence in the posterior and anterior hippocampus, the BLA, CMA, vmPFC, and dorsal lateral prefrontal cortex. The decision making concept cluster shows robust VMPFC, striatal (e.g., caudate), and sgACC activation. The cognition concept cluster shows a hippocampal and frontoparietal neural profile, including the middle frontal gyrus (MFG) in addition to the dorsal subdivision of the anterior insula. An “independent” category was used to capture terms that were not strongly associated with the four conceptual clusters of meaning. Since the “independent” category was diffuse with minimal within-category association (see Fig. 2C), we did not create a brain map to represent this category. The terms that were independent include contextual, cognitive impairment, inhibition, response inhibition, and control.

**Table 1:** Statistical & network information related to the four clusters and “Independent” category.

Cluster	Count	Studies	Cluster size	PubMed hits	Degree	Betweenness	Page rank
Cognition	9	1268.9	4758.9	2333.6	4.67	59.113	0.0245
Emotion	16	621.9	2730.3	11868.5	8.44	53.695	0.0282
Memory Function	6	464.7	9252.0	6908.8	6.33	103.633	0.0257
Independent	5	1013.4	1360.6	2733.0	0.40	0.000	0.0127
Decision Making	5	712.6	3981.4	1669.6	5.40	40.612	0.0223

Graph metrics of degree, betweenness, and page rank report the weighted non-binarized graph analysis.

Table 1 depicts statistical information related to the four conceptual clusters and “the independent” category, including the number of terms included in each cluster, number of studies associated with each cluster, cluster size, number of PubMed results yielded, and degree, closeness, betweenness, and page rank values. Notably, the emotion and memory functioning clusters have the highest levels of betweenness and degree. Table 2 depicts whole-brain correlations between the four conceptual clusters; these results further reinforce the relative independence of the four clusters. The Neurosynth term ‘PTSD’ was also examined even though it did not meet our inclusion threshold (‘PTSD’ was the 906th term), and it was too sparse (active voxels = 159; contributing studies = 106) to provide a coherent map that would show meaningful relationships with the other terms and clusters reported here. Indeed, the Neurosynth ‘PTSD’ term was weakly correlated with the concept cluster maps we identified (*rs* $\:\le\:$ 0.087; see Table 2).

**Table 2:** Correlations between Neurosynth ‘PTSD’ and four meta-analytic cluster brain maps.

Cluster	Cognition	Decision making	Emotion	Memory function
PTSD	−0.001	0.005	0.086	0.008
Cognition		0.014	−0.055	0.372
Decision Making			0.136	0.029
Emotion				0.117

Correlations across PTSD term map and construct cluster were computed using whole brain maps.

**Fig. 3:**
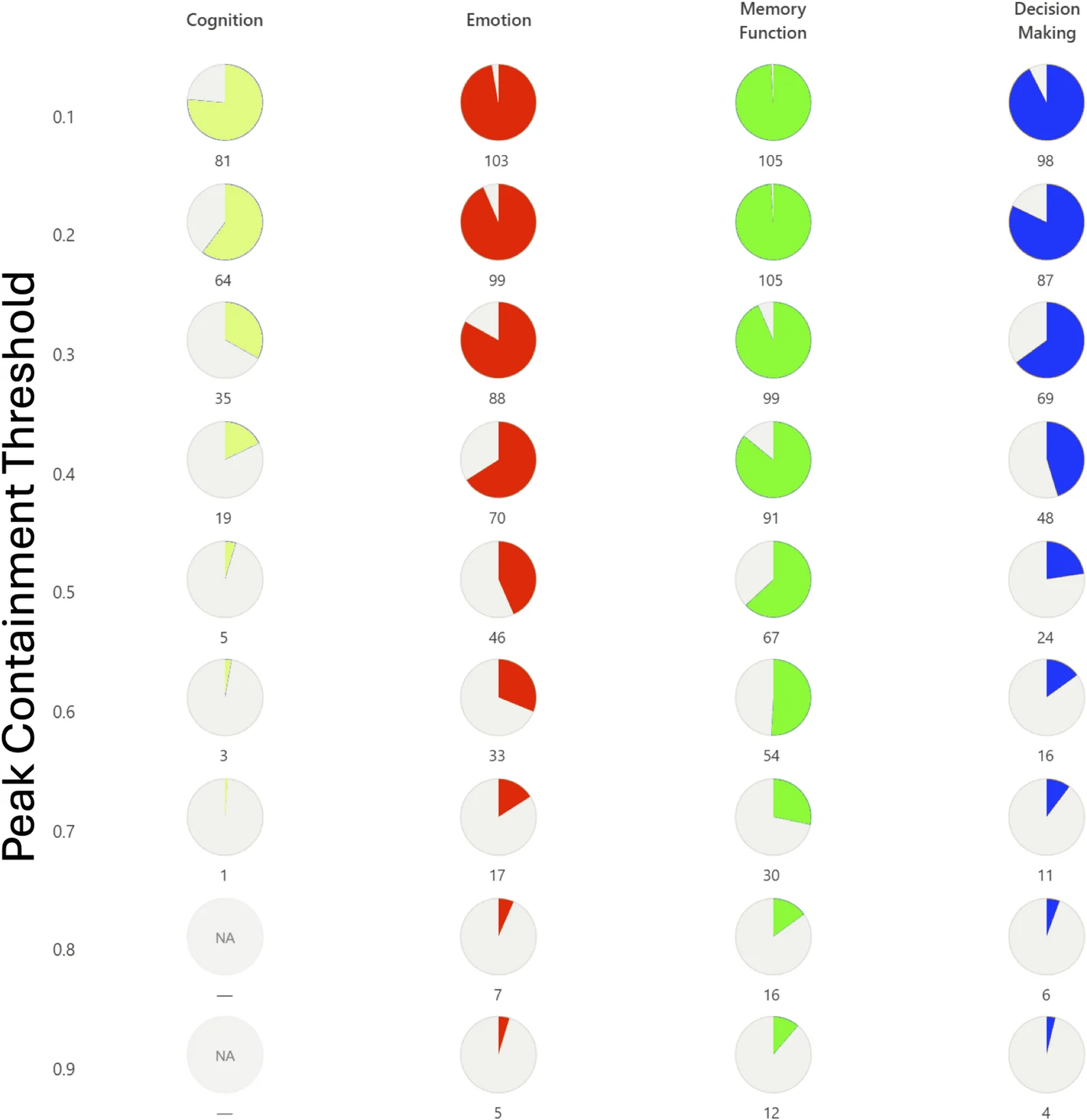
Article-specific ‘PTSD’ brain map containment within Concept Brain Networks. Article specific brain maps were created from the 106 papers comprising the Neurosynth “PTSD” term to determine whether brain regions were represented within each of the four identified Concept Brain Networks. For each article-specific map, peak containment was defined as the proportion of coordinate-derived ROIs that intersected a given Concept Brain Network. The y-axis shows the minimum peak-containment threshold, ranging from 0.10 to 0.90. Values below each pie chart indicate the number of PTSD studies meeting or exceeding that threshold for each network. Colored wedges indicate the proportion of studies represented within the corresponding Concept Brain Network, whereas gray wedges indicate studies not meeting the threshold. As shown, PTSD-related coordinates were differentially represented across the four Concept Brain Networks, with the strongest and most persistent containment observed for the Memory Function and Emotion networks.

To further investigate the link between the conceptual clusters and the PTSD Neurosynth term, we conducted two secondary analyses to evaluate how the derived Concept Brain Network maps related to the Neurosynth ‘PTSD’ term (see Methods for details). First, we tested the extent to which ROIs from the 106 individual studies comprising the Neurosynth PTSD term were contained within each of the four concept networks. This analysis revealed that the brain regions from the studies that comprise the PTSD Neurosynth term are differentially represented across the four conceptual networks (see Fig. 3). Across peak-containment thresholds, study-specific coordinates showed the strongest representation within the Memory functioning and Emotion conceptual clusters, while cognition and decision making had the lowest overall representation. Second, we added the PTSD term map to the original 41 Neurosynth terms and conducted an identical clustering analysis to determine where it fell with the broader construct organization. This result indicated that the PTSD term fell into the Emotion concept cluster, likely because the Neurosynth PTSD term consists primarily of amygdala activation. created article-specific brain maps for each of the 106 papers and examined whether brain regions from each individual paper comprising the PTSD Neurosynth term overlapped with any of the four conceptual clusters that we identified (see Methods).

Next, we examined the spatial correspondence between the four Concept Brain Network maps and established neural network maps, including salience, executive control, sensorimotor, default mode, and visual networks. The strongest correspondence was observed between the Cognition Concept Brain Network and the ECN-C and ECN-NS resting-state maps (see Table 3). In contrast, the Decision Making Concept Brain Network showed low correspondence with canonical resting-state networks from both the CAREN Atlas and Neurosynth-derived terms (*r*s < 0.096), suggesting that neural circuits associated with decision making and reward learning are not well captured by these canonical network definitions. We then conducted article-specific mapping of the Neurosynth “PTSD” term to examine whether PTSD study coordinates were represented within the canonical resting-state networks. Overall, PTSD study coordinates showed lower representation within the canonical resting-state networks than within the Concept Brain Networks (see Supplementary Figs. 2 and 3).

**Table 3:** Correlations between the four conceptual clusters and canonical resting state networks.

Cluster	CAREN networks					Neurosynth networks		
	SN-C	ECN-C	SMN-C	DMN-C	VIS-C	SN-NS	ECN-NS	DMN-NS
Cognition	0.031	0.319	0.002	0.068	0.027	0.209	0.529	0.053
Decision Making	0.006	0.010	−0.002	0.022	−0.002	0.095	0.063	0.033
Emotion	0.018	−0.023	−0.007	0.084	0.001	0.130	−0.048	0.080
Memory Function	0.004	0.078	0.000	0.202	0.074	0.130	0.122	0.231

SN-C, Salience network from CAREN Atlas; ECN-C, Executive Control Network from CAREN Atlas; SMN-C, Sensorimotor network from CAREN Atlas; DMN-C, Default mode network from CAREN Atlas; VIS-C, Visual network from CAREN Atlas; SN-NS, Salience network from NeuroSynth; ECN-NS, Executive control network from NeuroSynth; DMN-NS, Default mode network from Neurosynth.

## Discussion

The goal of this study was to address a critical barrier in PTSD research: the fragmentation of psychological constructs and corresponding neural mechanisms due to inconsistent terminology. To do so, we applied a clustering approach to Neurosynth term-based neural maps generated from a normative meta-analytic framework, after filtering terms for PTSD relevance using PubMed text-mining. Terms were prioritized based on their frequency of use in PTSD and PTSD+fMRI publications, allowing us to identify constructs that are meaningfully represented in PTSD research discourse. This approach organized PTSD-relevant psychological constructs into four broad clusters: cognition, emotion, memory functioning, and decision making. These clusters should be interpreted as neuroimaging-informed cluster maps derived from the broader neuroimaging literature, rather than as evidence of PTSD-related deficits, dysfunction, or pathophysiology. Accordingly, the present findings do not establish that individuals with PTSD show impairments in each cluster, nor do they indicate that the associated neural maps reflect PTSD-specific abnormalities. Instead, this framework provides a heuristic for organizing the heterogeneous terminology used across PTSD research and for interpreting PTSD neuroimaging findings in relation to broader psychological construct domains and their associated patterns of co-activation. The present approach may help bridge meta-analytic construct mapping with connectome-based models of brain organization while supporting more consistent terminology and hypothesis generation in PTSD research (see Supplementary Fig. 4 for a conceptual link between our results and DSM-5 based PTSD construction). We next consider how each concept cluster organizes PTSD-relevant constructs in relation to existing PTSD neuroimaging and clinical literature.

The emotion cluster included terms related to fear and threat processing, including fear, aversive, reactivity; heightened physiological and affective activation, including arousal, stress, and anxiety; and broader mood- and affect-related processes, including affect, regulation, mood, depression. Anchored in the CMA, BLA, sgACC, vmPFC, and dACC, this cluster therefore captures a broad affective construct space. Importantly, this cluster should not be interpreted as evidence of PTSD-specific dysfunction within these regions, but rather as a normative meta-analytic map of emotion-related constructs frequently investigated in PTSD research. This interpretation is consistent with PTSD-specific neuroimaging investigations showing heightened amygdala responsivity with reduced vmPFC/rostral ACC engagement during threat, extinction, and emotion regulation^14,38,39^. Amygdala hyper-reactivity has also been identified as a potential risk factor for developing PTSD^40,41^. Meta-analytic work further supports this pattern of greater limbic reactivity when processing negative emotions in PTSD^13,42^. Thus, although PTSD studies often use heterogenous terminology to describe fear, arousal, stress, mood, and emotion regulation processes, the present findings suggest that these constructs may converge at the neural systems level within a broader domain-general emotion circuit centered on amygdala-vmPFC-cingulate network.

The memory functioning cluster included terms related to core mnemonic processes, including memory performance, episodic memory, retrieval, encoding, memories, and personal. Anatomically, this cluster was anchored in the anterior and posterior hippocampus, BLA, CMA, vmPFC, and areas of the dorsolateral prefrontal cortex, consistent with a broad construct domain that has long been central to conceptualizations of PTSD as a “disorder of memory”^43–45^. Like with the emotion cluster, this map should not be interpreted as direct evidence of PTSD-specific memory dysfunction. Rather, it suggests that heterogeneous memory-related constructs converge onto a domain-general neural architecture supporting episodic encoding, retrieval, and autobiographical memory, In PTSD specific research, alterations in memory functioning is associated with persistent intrusive recollections and fragmented trauma memories that disrupt psychological, behavioral, and social functioning long after exposure^46–48^, with avoidance of trauma reminders further shaping daily behavior and decision-making^49^. Experimental work further links PTSD to impairments in extinction recall and contextual processing^8^, emphasizing the role of the hippocampus, amygdala, and medial prefrontal regions in contextualizing threat and regulating retrieval of traumatic memories^6,15^. Structural and functional abnormalities in the hippocampus contribute to memory fragmentation and overgeneralization of threat^50,51^, amplifying vulnerability to intrusive recall. In parallel, dlPFC involvement reflects the demands of working memory and top-down cognitive control during the recollection and regulation of trauma-related memories^6,15^. Take together, the memory functioning cluster provides a neuroimaging-informed framework for linking episodic memory encoding and retrieval within a shared hippocampal-amygdala-prefrontal network relevant to long-term functioning following trauma.

The decision-making cluster is consistent with a broad construct domain linking terms related to decision, reward, learning, loss, and anticipation and was centered on vmPFC, caudate nucleus of the striatum, sgACC, and CMA circuitry. Within this broader psychological construct framework, the decision-making cluster highlights a valuation and motivational learning domain in which reward anticipation, loss, processing, and approach-avoidance decisions are organized around vmPFC-striatal circuitry. This interpretation is clinically relevant given that anhedonia is common PTSD and is associated with increased functional and psychosocial impairment in PTSD patients^52,53^. Factor analytic studies further support diminished interest, detachment, and inability to experience positive emotions as a distinct symptom PTSD-related symptom^54,55^. Network analyses further suggest that anhedonia symptoms often occupy central positions within PTSD symptom networks, where they may exacerbate or maintain other symptom domains^56^. Despite this clinical importance, reward-related decision making processes remain relatively understudied in PTSD compared to threat and fear circuitry^57^. Converging neuroimaging evidence supports the relevance of anhedonia-related reward impairments in PTSD^58^, with reduced striatal and OFC responsivity to monetary, social, and natural rewards and altered reinforcement learning signals linked to emotional numbing and withdrawal^11^. Collectively, these decision-making cluster provides a neuroimaging-informed framework for linking learning, reward, loss processing within a shared vmPFC-striatal circuit.

The cognition cluster included terms related to attention, working memory, executive control, cognitive control, and interference, and was expressed primarily in the hippocampal and frontoparietal network but also included the CMA, BLA, and the dorsal portion of the anterior insula. This pattern is consistent with a broad cognitive-control domain linking attentional allocation, working memory demands, interference resolution, and executive regulation. Recent cognitive and neuroimaging reviews highlight impairment in selective attention, working memory, and cognitive flexibility, often magnified by trauma-relevant cues, with corresponding hypo- or hyperactivation across dorsal attention and frontoparietal control networks^10,15,59–61^. Meta-analytic work on threat-related attentional bias further underscores the role of biased orienting and impaired disengagement in the etiology and persistence of PTSD^7,62^. These cognitive processes may also perpetuate hypervigilance and avoidance but also compete with processing of safety and reward cues, thereby reinforcing anhedonia and motivational withdrawal. From a large-scale network perspective, this cluster aligns with central executive and salience network dynamics that shape vigilance, interference resolution, and cognitive performance under trauma-related distraction^63,64^. Importantly, interventions have begun to directly target these cognitive processes. Attention bias modification training (ABMT) shows mixed but encouraging effects in reducing hypervigilance and improving threat disengagement^65,66^, while working memory training protocols designed to strengthen prefrontal control have demonstrated preliminary efficacy in reducing threat bias and enhancing emotion regulation in trauma-exposed and anxious samples^67,68^. Collectively, the cognition cluster provides a neuroimaging-informed framework for linking attention, working memory, and executive control within frontoparietal-salience circuitry, processes relevant to PTSD.

The “independent” category included terms related to contextual, cognitive impairment, inhibition, response inhibition, and control. Although these terms are clearly relevant to PTSD research (as evidenced by the mean PubMed hits), they did not cluster with one of the four primary Concept clusters based on spatial similarity of their whole-brain meta-analytic Neurosynth maps. This highlights an important distinction between conceptual relatedness and neural-map similarity: terms that appear semantically related to a broader construct domain may show distinct, more distributed, or more task-dependent patterns of neural activation^69^. For example, although ‘contextual’ is conceptually related to memory functioning, its neural map may span hippocampal-prefrontal systems involved in memory functioning, but as well as broader processes related to salience detection and environmental monitoring. Thus, the independent category does not indicate that these constructs are peripheral to PTSD, rather these terms may identify PTSD-relevant psychological constructs whose neural representations are more cross-domain, multidimensional, or spatially distinct from the primary co-activation patterns captured by the other four conceptual clusters.

Emotion and memory functioning emerged as the most central psychological concept domains, showing the highest betweenness and strongest representation across the 106 studies comprising the Neurosynth ‘PTSD’ term (Fig. 3). These domains may serve as central organizing constructs or domains for PTSD neuroimaging discourse, linking constructs related to fear, arousal, regulation, episodic memory, encoding, retrieval, and personal memories. This pattern is consistent with longstanding models emphasizing PTSD as a disorder involving altered threat processing and trauma memory, including interactions among amygdala, hippocampal, medial prefrontal, and anterior cingulate cortex^6^. The concept clusters identified in the current investigation suggest that heterogeneous terms used across PTSD studies may converge onto broader domain-general emotion and memory functioning systems. A testable hypothesis arising from these findings is that PTSD symptom heterogeneity may be partly organized by the degree to which the neural circuits underlying emotion and memory functioning interact^70^.

The decision-making cluster demonstrated relatively high spatial ROI peak containment to the brain maps from the 106 studies comprising the PTSD Neurosynth term relative to the cognition concept cluster, but less than emotion and memory functioning. Despite having relatively high PubMed counts, this suggests that decision-making and reward learning literature may appear less developed in PTSD neuroimaging studies than the more established literatures on terms comprising emotion and memory functioning^57^. Consistent with this interpretation, research on examining reward-processing and anhedonia in PTSD remain relatively limited, often use small samples, and vary substantially in task design^11,58^. Clearly, further research is needed to examine how reward learning and decision-making processes are altered in PTSD.

We also examined the relationship between our four identified conceptual clusters and the core PTSD resting-state networks^64,71^: DMN, SN, and CEN. The highest association was observed between the cognition conceptual cluster and the ECN-C and ECN-NS resting state networks. In agreement with these findings, it has been shown that the CEN, anchored in dorsolateral prefrontal cortex and posterior parietal cortex, is responsible for high-level cognitive functions, including the control of attention and working memory^72^. However, the goal of the present analysis was not to recover canonical resting-state networks, but to organize PTSD-relevant psychological constructs based on whole-brain meta-analytic activation patterns. Therefore, weaker correspondence between some concept clusters, particularly the decision-making concept cluster, and resting-state networks should not be interpreted as problematic. Instead, it may indicate that the task-derived construct maps capture psychological processes that are not fully represented by broad resting-state networks. Although the salience network has extensive connectivity with subcortical and limbic regions involved in decision-making, reward, and motivation^12,73^, the low association between the decision-making concept cluster with resting-state maps suggests that reward-related task activations may reflect circuit configurations that are more context-dependent than those captured by canonical resting-state networks. This raises the hypothesis that PTSD-relevant reward and decision-making abnormalities may be better characterized using task-based or computational fMRI paradigms than resting-state networks^74^.

These findings highlight how the present concept clustering approach complements established network-identification methods. Independent component analysis (ICA) and related resting-state parcellation methods have been instrumental in defining the brain’s intrinsic network architecture by identifying temporally coherent systems, such as default mode, salience, and executive control networks^75–77^. However, these methods are optimized to characterize shared signal covariance at rest rather than the psychological constructs that guide task-based research^78^. In contrast, the present approach clusters whole-brain meta-analytic maps associated with psychological terms, thereby organizing constructs according to distributed patterns of co-activation rather than resting-state covariance. Specifically, the 41 Neurosynth term maps were selected because the corresponding psychological terms were frequently co-mentioned in PubMed articles containing “PTSD + fMRI,” identifying the concepts commonly studied in the PTSD neuroimaging. The resulting conceptual maps therefore represent the broader cognitive–affective construct domains that organize PTSD research, such as cognition, emotion, memory functioning, and decision-making, rather than PTSD-specific activation patterns derived from patient cohorts. This design situates our approach between meta-analytic construct mapping and connectome-based parcellation frameworks. Whereas network-based parcellations delineate the brain’s intrinsic architecture^75,76,78^, our method defines psychologically meaningful axes within that architecture^33^. In doing so, construct-based mapping provides a heuristic for interpreting PTSD neuroimaging findings by linking regional activation to broader constellations of co-activated regions associate with conceptually related constructs. This bridges large-scale functional systems with conceptual dimensions most relevant to trauma-related psychopathology.

A related strength of this approach is that it illustrates how individual brain regions can contribute to multiple conceptual domains, rather than mapping exclusively onto a single psychological construct or network. For example, the BLA, CMA, and vmPFC were prominent ROIs across multiple conceptual clusters. The regional overlap may reflect the functional versatility of these regions, consistent with evidence that certain neural areas act as flexible hubs that dynamically reconfigure across contexts to support diverse cognitive–affective–behavioral functions^79–81^. This overlap may also arise because psychological dimensions, such as salience, arousal, or valuation, cut across multiple higher-order domains including emotion, cognition, and memory functioning, decision-making. These findings highlight a central feature of the human brain: both the brain and the psychological constructs used to study it are organized through overlapping, context-dependent systems rather than discrete networks, an idea increasingly recognized in contemporary models of cognitive neuroscience^82^.

## Limitations

A limitation of the present study is that Neurosynth term-based maps are derived from studies across diverse populations and experimental contexts, rather than from PTSD samples alone. Accordingly, the four identified conceptual clusters should not be interpreted as PTSD-specific neural abnormalities. Instead, they represent neuroimaging-informed concept maps for psychological terms that are relevant to PTSD research discourse. This approach is intentional because the goal was to leverage the broader task-based neuroimaging literature to organize constructs commonly studied in PTSD, while using PubMed “PTSD” and “PTSD+fMRI” searches to verify the relevance of the selected terms to PTSD research. We further evaluated PTSD relevance by creating article-specific brain maps from the 106 studies comprising the Neurosynth “PTSD” term and testing whether these study maps overlapped with the four identified concept clusters. A second limitation is that the term-selection procedure included author ratings and imposed conceptual and statistical boundaries on the construct space examined. The initial focus on the top 600 Neurosynth terms necessarily excluded less frequent or more specific terms that may nevertheless be relevant to PTSD. Thus, our approach was not a fully unconstrained, data-driven analysis of all possible constructs, but rather to define a broad and interpretable construct space with adequate meta-analytic support. This cutoff was selected to balance breadth against inclusion of terms with sparse contributing studies or terms that were increasingly anatomical, methodological, or difficult to interpret. Future work should directly test whether these construct networks are altered in PTSD samples, and whether they provide greater explanatory value for task-based neuroimaging than canonical resting-state networks such as DMN, SN, and CEN. Another important direction is to evaluate whether these construct maps differ across clinically relevant subgroups. For example, PTSD and mild traumatic brain injury (mTBI) often co-occur^83^, and prior meta-analytic findings suggest convergence between PTSD and mTBI in the middle frontal gyrus, which is part of the cognition concept cluster^84^. Comparing concept cluster organization in PTSD with and without comorbid mTBI may therefore clarify whether truma-related psychopathology and neurotrauma differentially engage cognition, memory functioning, emotion, and decision-making systems.

In conclusion, these findings demonstrate that PTSD-relevant psychological constructs can be organized into four neuroimaging-informed conceptual domains: cognition, emotion, memory functioning, and decision-making, each associated with distinct but overlapping patterns of neural architecture. This framework supports the view that PTSD is unlikely to have a single unified neural representation; rather PTSD may be better understood as an emergent clinical construct shaped by interacting cognitive-affective mechanisms. By linking heterogeneous terminology in the PTSD literature to broader construct level neural systems, the present approach provides a heuristic for interpreting neuroimaging findings, reducing conceptual fragmentations, and generating mechanistic hypotheses about the processes that collectively contribute to PTSD.

## Method

See Supplementary Fig. 1 for a flow chart depicting the analysis plan.

### Selection of Neurosynth terms

First, we evaluated the distribution of contributing studies across 3,228 Neurosynth terms to assess whether the initial 600-term cutoff balanced construct breadth with adequate meta-analytic support. An elbow-based cutoff suggests a more restrictive threshold of 872 contributing studies, which would have retained only 221 terms (see Supplementary Fig. 5). We had also examined an elbow-cutoff based on the “PTSD” and “PTSD+fMRI” PubMed searches (“all fields” and “MeSH terms”). The elbow-based thresholds were 485 terms for PTSD-related publications and 613 terms for PTSD+fMRI publications (see Supplementary Fig. 6). Applying all three possible frequency criteria would have retained only 75 terms, which we considered too narrow for the aim of clustering broad PTSD-relevant psychological constructs. Therefore, we chose to select the top 600 terms to preserve a broader candidate construct space while excluding increasingly sparse, methodological, or difficult to interpret terms. Second, the three authors (RSG, ANS, DMS) independently categorized the terms as “brain” (describes a brain region), “construct” (describes a property of human behavior), or “other” (describes either a property of behavior, analysis, or brain — uncertain). The majority-selected (2 of 3 or 3 of 3 raters) categorization was then used (brain: *n* = 186; construct: *n* = 177; other: *n* = 237). Interrater reliability was high: Fleiss’s Kappa = 0.88, Z = 52.4, *p* < 0.0001.

### Linking terms to PTSD

Subsequently, the three authors independently selected terms from the construct category related to PTSD (Fleiss’s Kappa = 0.72, Z = 16.5, *p* < 0.0001). Here PTSD-relevant terms are defined as psychological constructs that are meaningfully represented in PTSD research discourse and may help organize the psychological domains commonly examined in the field. This does not imply that these constructs are PTSD-specific where there is evidence of unique alterations or directly indicative of PTSD-related neural dysfunction. If the majority of authors endorsed the term as relating to PTSD, the term was retained, yielding 69 PTSD-related construct terms. In order to more objectively verify the selections are PTSD-relevant, each term was filtered through PubMed searches with the terms “PTSD” and “PTSD+fMRI” (“all fields” and “MeSH terms” options were selected). The resulting terms should be interpreted as neuroimaging-informed construct maps relevant to PTSD research, not PTSD-specific abnormalities.

Drop rules were then developed to determine which of the 69 terms would be discarded (see Supplementary Table 2 for details). Rules were created based on data gathered from Neurosynth and PubMed, and redundancy was also considered. Neurosynth drop rules were the following: drop the term if 2 of the 3 are true: (1) if max *r* of a Neurosynth map against other term maps < 0.1, (2) if median *r* of a Neurosynth map against other term maps = 0, (3) if voxel count < 100. The aim of these drop rules was to remove small or outlier Neurosynth concepts. PubMed drop rules were the following: (1) if below 200 papers in PubMed with the “PTSD” search, (2) if below 25 papers in PubMed with the “PTSD+fMRI” search. Redundancy drop rules were the following: contains same word and satisfies either: (a) if *r* > 0.5 correlation with other term, or (b) concepts with added “(s)”. When redundant concepts were identified, we chose the term that had the most Neurosynth studies contributing. These rules resulted in 28 dropped terms, leaving 41 terms related to PTSD.

### Clustering brain maps

Whole-brain Pearson correlations were computed between each pair of Neurosynth term-based brain maps using AFNI’s *3ddot*, and the resulting unique pairwise correlations were ranked. To determine an appropriate threshold for constructing the binary network graph, we evaluated a range of candidate cutoffs retaining the top 5% to 25% of correlations and compared metrics (i.e., classification accuracy, Jaccard similarity, Dice similarity, and chi-square) across thresholds (see Supplementary Fig. 7). Based on the overall pattern of these metrics, the top 15% threshold provided the best balance and was selected for subsequent analyses. Threshold stability was evaluated across all candidate thresholds using 500 bootstrap iterations and three network-perturbation procedures: subset resampling (a random draw of ¾ of edges were retained), edge drop (10% of edges were removed), and jitter (5% of node connections are deleted and replaced with new connections). For each perturbed solution, we quantified clustering consistency using four complementary metrics: Jaccard similarity, adjusted Rand index (ARI), normalized mutual information (NMI), and variation of information (VI). Results showed that the confidence intervals substantially overlapped across most thresholds and metrics (see Supplementary Fig. 8), indicating that clustering stability was robust across a range of plausible thresholds rather than being dependent on a single cutoff. Stability statistics for the chosen 15% threshold is reported in Supplementary Table 4. We also report individual term stability across the tested thresholds in Supplementary Fig. 9.

Correlations above threshold were retained to construct a network graph, which then was clustered using fast-greedy community detection (Clauset et al., 2004). See Supplementary Figs. 10 and 11 for binarized heatmap of Neurosynth terms and graph network plots. This approach allowed for the identification of clusters of meaning among terms. Statistical information, including metrics such as degree, betweenness, and page rank, were exported along with the correlation heatmaps and construct graph maps.

### Linking concept maps to Neurosynth PTSD term

We examined the relationship between the Concept Brain Network maps with the ‘PTSD’ Neurosynth term in two ways. First, we created article-specific brain maps from the papers comprising the Neurosynth ‘PTSD’ term (*n* = 106 studies). Next, we extracted the PubMed identification numbers (IDs) for all 106 papers associated with the Neurosynth meta-analysis term ‘PTSD’ and extracted the *x*,* y*,*z* coordinates for each brain region reported in each study. As an initial preprocessing step, we evaluated spherical ROI radii ranging between 3 and 9 mm around each reported coordinate and compared the resulting study-specific maps with the Concept Brain Network maps across multiple spatial association metrics (correlation, dice coefficient, Jaccard index; see Supplementary Fig. 12). The 6-mm radius was selected because it maximized the number of links between PTSD study-specific brain maps and the Concept Brain Network maps, while also matching the radius used by Neurosynth. Using the selected 6-mm radius, we then created spherical ROIs around each coordinate within each study, yielding 106 study-specific PTSD brain maps. For the primary validation analysis, we did not use Dice or Jaccard similarity because these symmetric overlap metrics are most appropriate for comparing two binary maps of comparable spatial extent. In the present case, the comparison involved sparse coordinate-derived ROIs and spatially extended Concept Brain Network maps; therefore, Dice and Jaccard values would penalize the size mismatch between point maps and networks and are sensitive to the chosen sphere radius. Instead, we quantified permutation-tested asymmetric peak containment, defined as the proportion of ROIs from each PTSD study-specific brain map that intersected a given Concept Brain Network map, independent of network size. For example, if 4 ROIs from a study-specific map containing 16 ROIs intersected the Attention Network, the peak-containment value was 0.25. Higher values therefore indicate that a greater proportion of coordinates from a given PTSD study fell within a Concept Brain Network. We then examined the number of PTSD studies showing peak-containment values with each Concept Brain Network across a range of containment thresholds from 0.1 to 1.0. A larger number of studies exceeding a given threshold indicates that coordinates from the PTSD literature were more consistently represented within that Concept Brain Network.

Second, we examined how the Neurosynth ‘PTSD’ term map related to the broader construct clustering analysis. The PTSD term was not included in the primary analysis because the primary aim was to organize psychological and mechanistic construct terms, whereas PTSD represents a disorder label rather than a psychological mechanism. However, to determine where the PTSD term map would fall within the derived construct structure, we conducted a secondary clustering analysis in which the Neurosynth “PTSD” term map was added to the set of retained construct maps. The PTSD map was incorporated into the threshold binary graph using the same procedures described in our primary analysis and submitted to the same fast-greedy community detection clustering.

### Linking concept maps to resting-state networks

Likewise, we examined the relationship between the concept clusters we identified and the core resting-state networks: DMN, SN, and CEN. This was done in two ways. First, we extracted brain maps for the visual (VIS-C), default mode (DMN-C), salience (SN-C), sensorimotor (SMN-C), and executive control (ECN-C) networks using the Consensual Atlas of Resting-state Networks (CAREN)^85^. This atlas is based on a hierarchical clustering of four common brain atlases to derive a set of five coherent resting-state networks. Second, we also downloaded the Neurosynth association maps of the default mode (DMN-NS), salience (SN-NS), and executive control (ECN-NS) networks. Using these two approaches, we correlated these different canonical resting-state network brain maps with our conceptual clusters to probe similarities and differences.

## Supplementary Information

Below is the link to the electronic supplementary material.

Supplementary Material 1

## Data Availability

The list of Neurosynth terms used to generate the term-based brain maps has been deposited with the analysis code in Zenodo and is available at https://doi.org/10.5281/zenodo.20834288. The term-based brain maps analyzed in this study were derived from Neurosynth using publicly available meta-analytic resources. The Neurosynth-derived brain maps are not redistributed with this manuscript but can be generated using the deposited term list and analysis workflow, subject to the availability and distribution terms of the Neurosynth resource.

## Code availability

The R scripts used to perform the analyses reported in this manuscript have been deposited in Zenodo and are available at https://doi.org/10.5281/zenodo.20834288. The repository includes the Neurosynth term list used to generate the term-based brain maps, analysis scripts, and documentation supporting reproduction of the reported statistical analyses from the derived inputs.

## References

[1] Goldstein, R. B. et al. The epidemiology of DSM-5 posttraumatic stress disorder in the United States: Results from the National Epidemiologic Survey on Alcohol and Related Conditions-III. *Soc. Psychiatry Psychiatr Epidemiol.* **51**, 1137–1148. 10.1007/s00127-016-1208-5 (2016). PMC4980174 27106853

[2] Boscarino, J. A. Posttraumatic stress disorder and mortality among U.S. Army veterans 30 years after military service. *Ann. Epidemiol.* **16**, 248–256. 10.1016/j.annepidem.2005.03.009 (2006). 16099672

[3] Association, A. P. *Diagnostic and Statistical Manual of Mental Disorders*. 5th edn, (2013).

[4] Hundt, N. E. et al. It didn’t fit for me: A qualitative examination of dropout from prolonged exposure and cognitive processing therapy in veterans. *Psychol. Serv.* **17**, 414–421. 10.1037/ser0000316 (2020). PMC6535373 30475041

[5] Steenkamp, M. M. True evidence-based care for posttraumatic stress disorder in military personnel and veterans. *JAMA Psychiatry*. **73**, 431–432. 10.1001/jamapsychiatry.2015.2879 (2016). 26886378

[6] Liberzon, I. Searching for intermediate phenotypes in posttraumatic stress disorder. *Biol. Psychiatry*. **83**, 797–799. 10.1016/j.biopsych.2017.06.005 (2018). 28648648

[7] Bar-Haim, Y., Lamy, D., Pergamin, L. & Bakermans-Kranenburg, M. J. Threat-related attentional bias in anxious and nonanxious individuals: A meta-analytic study. *Psychol. Bull.* **133**, 1–24. 10.1037/0033-2909.133.1.1 (2007). 17201568

[8] Milad, M. R. et al. Neurobiological basis of failure to recall extinction memory in posttraumatic stress disorder. *Biol. Psychiatry*. **66**, 1075–1082. 10.1016/j.biopsych.2009.06.026 (2009). PMC2787650 19748076

[9] Acheson, D. T. et al. A prospective study of pre-trauma fear learning and extinction as risk factors for posttraumatic stress disorder. *J. Mood Anxiety Disord*. **12**, 100148. 10.1016/j.xjmad.2025.100148 (2025). PMC12524147 41103636

[10] Aupperle, R. L., Melrose, A. J., Stein, M. B. & Paulus, M. P. Executive function and PTSD: Disengaging from trauma. *Neuropharmacology* **62**, 686–694. 10.1016/j.neuropharm.2011.02.008 (2012). PMC4719148 21349277

[11] Vinograd, M., Stout, D. M. & Risbrough, V. B. in *Current Topics in Behavioral Neurosciences* 1–15 (Springer Berlin Heidelberg, 2022). 10.1007/7854_2021_292 34907507

[12] Menon, V. & Uddin, L. Q. Saliency, switching, attention and control: A network model of insula function. *Brain Struct. Funct.* **214**, 655–667. 10.1007/s00429-010-0262-0 (2010). PMC2899886 20512370

[13] Etkin, A. & Wager, T. D. Functional neuroimaging of anxiety: A meta-analysis of emotional processing in PTSD, social anxiety disorder, and specific phobia. *Am. J. Psychiatry*. **164**, 1476–1488. 10.1176/appi.ajp.2007.07030504 (2007). PMC3318959 17898336

[14] Liberzon, I. & Sripada, C. S. The functional neuroanatomy of PTSD: A critical review. *Prog. Brain Res.* **167**, 151–169. 10.1016/S0079-6123(07)67011-3 (2008). 18037013

[15] Hayes, J. P., Vanelzakker, M. B. & Shin, L. M. Emotion and cognition interactions in PTSD: A review of neurocognitive and neuroimaging studies. *Front. Integr. Neurosci.* **6**, 89. 10.3389/fnint.2012.00089 (2012). PMC3466464 23087624

[16] St Jacques, P. L., Botzung, A., Miles, A. & Rubin, D. C. Functional neuroimaging of emotionally intense autobiographical memories in post-traumatic stress disorder. *J. Psychiatr. Res.* **45**, 630–637. 10.1016/j.jpsychires.2010.10.011 (2011). PMC3081954 21109253

[17] Nawijn, L. et al. Reward functioning in PTSD: A systematic review exploring the mechanisms underlying anhedonia. *Neurosci. Biobehav. Rev.* **51**, 189–204. 10.1016/j.neubiorev.2015.01.019 (2015). 25639225

[18] Elman, I. et al. Reward and aversion processing in patients with post-traumatic stress disorder: Functional neuroimaging with visual and thermal stimuli. *Transl. Psychiatry*. **8**, 240. 10.1038/s41398-018-0292-6 (2018). PMC6214971 30389908

[19] Burgoyne, A. P., Tsukahara, J. S., Mashburn, C. A., Pak, R. & Engle, R. W. Nature and measurement of attention control. *J. Exp. Psychol. Gen.* **152**, 2369–2402. 10.1037/xge0001408 (2023). 37079831

[20] Mackie, M. A., Van Dam, N. T. & Fan, J. Cognitive control and attentional functions. *Brain Cogn.* **82**, 301–312. 10.1016/j.bandc.2013.05.004 (2013). PMC3722267 23792472

[21] Menon, V. & D’Esposito, M. The role of PFC networks in cognitive control and executive function. *Neuropsychopharmacology* **47**, 90–103. 10.1038/s41386-021-01152-w (2022). PMC8616903 34408276

[22] Bouton, M. E. Extinction of instrumental (operant) learning: Interference, varieties of context, and mechanisms of contextual control. *Psychopharmacol. (Berl)*. **236**, 7–19. 10.1007/s00213-018-5076-4 (2019). PMC6374202 30350221

[23] Fullana, M. A. et al. Fear extinction in the human brain: A meta-analysis of fMRI studies in healthy participants. *Neurosci. Biobehav. Rev.* **88**, 16–25. 10.1016/j.neubiorev.2018.03.002 (2018). 29530516

[24] Lopresto, D., Schipper, P. & Homberg, J. R. Neural circuits and mechanisms involved in fear generalization: Implications for the pathophysiology and treatment of posttraumatic stress disorder. *Neurosci. Biobehav. Rev.* **60**, 31–42. 10.1016/j.neubiorev.2015.10.009 (2016). 26519776

[25] De Boeck, P. et al. Questioning psychological constructs: Current issues and proposed changes. *Psychol. Inq.* **34**, 239–257. 10.1080/1047840x.2023.2274429 (2024).

[26] Sullivan, J. A. Stabilizing constructs through collaboration across different research fields as a way to foster the integrative approach of the research domain criteria (RDoC) project. *Front. Hum. Neurosci.* **10**, 309. 10.3389/fnhum.2016.00309 (2016). PMC4923248 27445749

[27] Breslau, N., Reboussin, B. A., Anthony, J. C. & Storr, C. L. The structure of posttraumatic stress disorder: Latent class analysis in 2 community samples. *Arch. Gen. Psychiatry*. **62**, 1343–1351. 10.1001/archpsyc.62.12.1343 (2005). 16330722

[28] Redican, E. et al. The latent structure of ICD-11 posttraumatic stress disorder (PTSD) and complex PTSD in a general population sample from USA: A factor mixture modelling approach. *J. Anxiety Disord.* **85**, 102497. 10.1016/j.janxdis.2021.102497 (2022). 34785481

[29] Yarkoni, T., Poldrack, R. A., Nichols, T. E., Van Essen, D. C. & Wager, T. D. Large-scale automated synthesis of human functional neuroimaging data. *Nat. Methods*. **8**, 665–670. 10.1038/nmeth.1635 (2011). PMC3146590 21706013

[30] Eickhoff, S. B. et al. Coordinate-based activation likelihood estimation meta-analysis of neuroimaging data: A random-effects approach based on empirical estimates of spatial uncertainty. *Hum. Brain Mapp.* **30**, 2907–2926. 10.1002/hbm.20718 (2009). PMC2872071 19172646

[31] Dockès, J., Oudyk, K., Torabi, M., de la Vega, A. I. & Poline, J. B. (eLife Sciences, Ltd, 2024). 10.7554/eLife.94909 PMC12425474 40934113

[32] Poldrack, R. A. et al. Discovering relations between mind, brain, and mental disorders using topic mapping. *PLoS Comput. Biol.* **8**, e1002707. 10.1371/journal.pcbi.1002707 (2012). PMC3469446 23071428

[33] Alhazmi, F. H., Beaton, D. & Abdi, H. Semantically defined subdomains of functional neuroimaging literature and their corresponding brain regions. *Hum. Brain Mapp.* **39**, 2764–2776. 10.1002/hbm.24038 (2018). PMC6866474 29575246

[34] Hayes, J. P., Hayes, S. M. & Mikedis, A. M. Quantitative meta-analysis of neural activity in posttraumatic stress disorder. *Biol. Mood Anxiety Disord*. **2**, 9. 10.1186/2045-5380-2-9 (2012). PMC3430553 22738125

[35] Hinojosa, C. A., George, G. C. & Ben-Zion, Z. Neuroimaging of posttraumatic stress disorder in adults and youth: Progress over the last decade on three leading questions of the field. *Mol. Psychiatry*. **29**, 3223–3244. 10.1038/s41380-024-02558-w (2024). PMC11449801 38632413

[36] Xiao, S. et al. Functional and structural brain abnormalities in posttraumatic stress disorder: A multimodal meta-analysis of neuroimaging studies. *J. Psychiatr. Res.* **155**, 153–162. 10.1016/j.jpsychires.2022.08.010 (2022). 36029627

[37] Poldrack, R. A. et al. Scanning the horizon: Towards transparent and reproducible neuroimaging research. *Nat. Rev. Neurosci.* **18**, 115–126. 10.1038/nrn.2016.167 (2017). PMC6910649 28053326

[38] Pitman, R. K. et al. Biological studies of post-traumatic stress disorder. *Nat. Rev. Neurosci.* **13**, 769–787. 10.1038/nrn3339 (2012). PMC4951157 23047775

[39] Rauch, S. L., Shin, L. M. & Phelps, E. A. Neurocircuitry models of posttraumatic stress disorder and extinction: Human neuroimaging research–past, present, and future. *Biol. Psychiatry*. **60**, 376–382. 10.1016/j.biopsych.2006.06.004 (2006). 16919525

[40] Belleau, E. L. et al. Amygdala functional connectivity in the acute aftermath of trauma prospectively predicts severity of posttraumatic stress symptoms. *Neurobiol. Stress*. **12**, 100217. 10.1016/j.ynstr.2020.100217 (2020). PMC7231977 32435666

[41] de Voogd, L. D. et al. Amygdala hyperactivity in posttraumatic stress disorder: Disentangling predisposing from consequential factors using a prospective longitudinal design. *Biol. Psychiatry*. **98**, 427–435. 10.1016/j.biopsych.2025.02.894 (2025). 40024495

[42] Patel, R., Spreng, R. N., Shin, L. M. & Girard, T. A. Neurocircuitry models of posttraumatic stress disorder and beyond: A meta-analysis of functional neuroimaging studies. *Neurosci. Biobehav. Rev.* **36**, 2130–2142. 10.1016/j.neubiorev.2012.06.003 (2012). 22766141

[43] van Marle, H. PTSD as a memory disorder. *Eur. J. Psychotraumatology*. **6**, 103402ejptv340627633–103402ejpt2761023402. 10.3402/ejpt.v6.27633 (2015).

[44] Brewin, C. R. The nature and significance of memory disturbance in posttraumatic stress disorder. *Annu. Rev. Clin. Psychol.* **7**, 203–227. 10.1146/annurev-clinpsy-032210-104544 (2011). 21219190

[45] Desmedt, A., Marighetto, A. & Piazza, P. V. Abnormal fear memory as a model for posttraumatic stress disorder. *Biol. Psychiatry*. **78**, 290–297. 10.1016/j.biopsych.2015.06.017 (2015). 26238378

[46] Brewin, C. R. Memory and forgetting. *Curr. Psychiatry Rep.* **20**, 87. 10.1007/s11920-018-0950-7 (2018). PMC6132786 30155780

[47] Brewin, C. R., Gregory, J. D., Lipton, M. & Burgess, N. Intrusive images in psychological disorders: Characteristics, neural mechanisms, and treatment implications. *Psychol. Rev.* **117**, 210–232. 10.1037/a0018113 (2010). PMC2834572 20063969

[48] Clark, I. A. & Mackay, C. E. Mental imagery and post-traumatic stress disorder: A neuroimaging and experimental psychopathology approach to intrusive memories of trauma. *Front. Psychiatry*. **6**, 104. 10.3389/fpsyt.2015.00104 (2015). PMC4510312 26257660

[49] Iyadurai, L. et al. Intrusive memories of trauma: A target for research bridging cognitive science and its clinical application. *Clin. Psychol. Rev.* **69**, 67–82. 10.1016/j.cpr.2018.08.005 (2019). PMC6475651 30293686

[50] Karl, A., Schaefer, M., Malta, L. S., Dörfel, D., Rohleder, N. & Werner, A. A meta-analysis of structural brain abnormalities in PTSD. *Neurosci. Biobehav. Rev.* **30**(7), 1004–1031 (2006). 10.1016/j.neubiorev.2006.03.004 16730374

[51] Woon, F. L., Sood, S. & Hedges, D. W. Hippocampal volume deficits associated with exposure to psychological trauma and posttraumatic stress disorder in adults: A meta-analysis. *Prog. Neuropsychopharmacol. Biol. Psychiatry*. **34**, 1181–1188. 10.1016/j.pnpbp.2010.06.016 (2010). 20600466

[52] May, C. L., Wisco, B. E., Fox, V. A., Marx, B. P. & Keane, T. M. Posttraumatic stress disorder-related anhedonia as a predictor of psychosocial functional impairment among United States veterans. *J. Trauma. Stress*. **35**, 1334–1342. 10.1002/jts.22832 (2022). PMC9790620 35405033

[53] Miller, C. R., McDonald, J. E., Grau, P. P. & Wetterneck, C. T. Quality of life in posttraumatic stress disorder: The role of posttraumatic anhedonia and depressive symptoms in a treatment-seeking community sample. *Trauma. Care*. **4**, 87–97 (2024).

[54] Gentes, E. et al. Latent factor structure of DSM-5 posttraumatic stress disorder. *Psychopathol. Rev.* **2**, 17–29. 10.5127/pr.035914 (2015). PMC4563872 26366290

[55] Lee, D. J. et al. Latent factor structure of DSM-5 posttraumatic stress disorder: Evaluation of method variance and construct validity of novel symptom clusters. *Psychol. Assess.* **31**, 46–58. 10.1037/pas0000642 (2019). PMC6312504 30113182

[56] Contractor, A. A., Greene, T., Dolan, M., Weiss, N. H. & Armour, C. Relation between PTSD symptom clusters and positive memory characteristics: A network perspective. *J. Anxiety Disord*. **69**, 102157. 10.1016/j.janxdis.2019.102157 (2020). PMC6960352 31751918

[57] Stout, D. M. & Risbrough, V. B. Nothing ventured nothing gained: Understanding reward circuit mechanisms of posttraumatic stress disorder. *Biol. Psychiatry Cogn. Neurosci. Neuroimaging*. **8**, 877–879. 10.1016/j.bpsc.2023.07.009 (2023). 37678965

[58] Bennett, M. M., Davis, K. E. & Fitzgerald, J. M. Neural correlates of reward processing in the onset, maintenance, and treatment of posttraumatic stress disorder. *Biol. Psychiatry Cogn. Neurosci. Neuroimaging*. **8**, 884–890. 10.1016/j.bpsc.2023.05.007 (2023). 37263417

[59] Etkin, A. Vol. 2 (eds Murray B. Stein & Thomas Steckler) 251–277 (Springer Berlin Heidelberg, 2010).

[60] Ely, S. L. et al. Attention! Posttraumatic stress disorder is associated with altered attention-related brain function. *Front. Behav. Neurosci.* **17**, 1244685. 10.3389/fnbeh.2023.1244685 (2023). Attention. PMC10476007 37670803

[61] Aspelund, S. G. et al. Assessing neurocognitive outcomes in PTSD: A multilevel meta-analytical approach. *Eur. J. Psychotraumatol*. **16**, 2469978. 10.1080/20008066.2025.2469978 (2025). PMC11894747 40062977

[62] Cisler, J. M. & Koster, E. H. Mechanisms of attentional biases towards threat in anxiety disorders: An integrative review. *Clin. Psychol. Rev.* **30**, 203–216. 10.1016/j.cpr.2009.11.003 (2010). PMC2814889 20005616

[63] Menon, V. Large-scale brain networks and psychopathology: A unifying triple network model. *Trends Cogn. Sci.* **15**, 483–506. 10.1016/j.tics.2011.08.003 (2011). 21908230

[64] Akiki, T. J., Averill, C. L. & Abdallah, C. G. A Network-based neurobiological model of PTSD: Evidence from structural and functional neuroimaging studies. *Curr. Psychiatry Rep.* **19**, 81. 10.1007/s11920-017-0840-4 (2017). PMC5960989 28924828

[65] Clauss, K. et al. When the attention control condition works: A systematic review of attention control training for posttraumatic stress disorder. *J. Trauma. Stress*. **38**, 16–28. 10.1002/jts.23104 (2025). 39365277

[66] Mogoase, C., David, D. & Koster, E. H. Clinical efficacy of attentional bias modification procedures: An updated meta-analysis. *J. Clin. Psychol.* **70**, 1133–1157. 10.1002/jclp.22081 (2014). 24652823

[67] Bomyea, J., Stein, M. B. & Lang, A. J. Interference control training for PTSD: A randomized controlled trial of a novel computer-based intervention. *J. Anxiety Disord*. **34**, 33–42. 10.1016/j.janxdis.2015.05.010 (2015). PMC4532583 26114901

[68] Schweizer, S. et al. Improving cognitive control in adolescents with post-traumatic stress disorder (PTSD). *Behav. Res. Ther.* **93**, 88–94. 10.1016/j.brat.2017.03.017 (2017). PMC5408907 28391116

[69] Poldrack, R. A. & Yarkoni, T. From brain maps to cognitive ontologies: Informatics and the search for mental structure. *Annu. Rev. Psychol.* **67**, 587–612. 10.1146/annurev-psych-122414-033729 (2016). PMC4701616 26393866

[70] Stout, D. M. et al. Multimodal canonical correlation reveals converging neural circuitry across trauma-related disorders of affect and cognition. *Neurobiol. Stress*. **9**, 241–250. 10.1016/j.ynstr.2018.09.006 (2018). PMC6234282 30450388

[71] Abdallah, C. G. et al. The neurobiology and pharmacotherapy of posttraumatic stress disorder. *Annu. Rev. Pharmacol. Toxicol.* **59**, 171–189. 10.1146/annurev-pharmtox-010818-021701 (2019). PMC6326888 30216745

[72] Bressler, S. L. & Menon, V. Large-scale brain networks in cognition: Emerging methods and principles. *Trends Cogn. Sci.* **14**, 277–290. 10.1016/j.tics.2010.04.004 (2010). 20493761

[73] Uddin, L. Q. Salience processing and insular cortical function and dysfunction. *Nat. Rev. Neurosci.* **16**, 55–61. 10.1038/nrn3857 (2015). 25406711

[74] Hiser, J. et al. Decision-making for concurrent reward and threat is differentially modulated by trauma exposure and PTSD symptom severity. *Behav. Res. Ther.* **167**, 104361. 10.1016/j.brat.2023.104361 (2023). PMC10370461 37393833

[75] Yeo, B. T. et al. The organization of the human cerebral cortex estimated by intrinsic functional connectivity. *J. Neurophysiol.* **106**, 1125–1165. 10.1152/jn.00338.2011 (2011). PMC3174820 21653723

[76] Power, J. D. et al. Functional network organization of the human brain. *Neuron* **72**, 665–678. 10.1016/j.neuron.2011.09.006 (2011). PMC3222858 22099467

[77] Betzel, R. F. et al. The community structure of functional brain networks exhibits scale-specific patterns of inter- and intra-subject variability. *Neuroimage* **202**, 115990. 10.1016/j.neuroimage.2019.07.003 (2019). PMC7734597 31291606

[78] Uddin, L. Q., Yeo, B. T. T. & Spreng, R. N. Towards a universal taxonomy of macro-scale functional human brain networks. *Brain Topogr*. **32**, 926–942. 10.1007/s10548-019-00744-6 (2019). PMC7325607 31707621

[79] Cole, M. W. et al. Multi-task connectivity reveals flexible hubs for adaptive task control. *Nat. Neurosci.* **16**, 1348–1355. 10.1038/nn.3470 (2013). PMC3758404 23892552

[80] Yuan, B. et al. The domain-separation language network dynamics in resting state support its flexible functional segregation and integration during language and speech processing. *Neuroimage* **274**, 120132. 10.1016/j.neuroimage.2023.120132 (2023). 37105337

[81] Shine, J. M. The thalamus integrates the macrosystems of the brain to facilitate complex, adaptive brain network dynamics. *Prog. Neurobiol.* **199**, 101951. 10.1016/j.pneurobio.2020.101951 (2021). 33189781

[82] Pessoa, L. The entangled brain. *J. Cogn. Neurosci.* **35**, 349–360. 10.1162/jocn_a_01908 (2023). PMC11019915 36007090

[83] Simonovic, M., Nedovic, B., Radisavljevic, M. & Stojanovic, N. The co-occurrence of post-traumatic stress disorder and depression in individuals with and without traumatic brain injury: A comprehensive investigation. *Med. (Kaunas)*. 10.3390/medicina59081467 (2023). PMC10456657 37629756

[84] Simmons, A. N. & Matthews, S. C. Neural circuitry of PTSD with or without mild traumatic brain injury: A meta-analysis. *Neuropharmacology* **62**, 598–606. 10.1016/j.neuropharm.2011.03.016 (2012). 21420986

[85] Doucet, G. E., Lee, W. H. & Frangou, S. Evaluation of the spatial variability in the major resting-state networks across human brain functional atlases. *Hum. Brain Mapp.* **40**, 4577–4587. 10.1002/hbm.24722 (2019). PMC6771873 31322303

