# Supplementary material for: A neuro-synthesis of psychological constructs relevant to posttraumatic stress disorder
Source: Sci Rep. 2026 Jul 9;16:31577. doi: 10.1038/s41598-026-60710-8 (PMC13649567; doi:10.1038/s41598-026-60710-8)
Supplement: Supplementary file 1 [file 41598_2026_60710_MOESM1_ESM.pdf]

Supplementary Material For:

A Neuro-Synthesis of Psychological Constructs Relevant to Posttraumatic Stress Disorder

Resh S. Gupta, Ph.D. [1]; Alan, N. Simmons, Ph.D.[2,3]; and Daniel M. Stout, Ph.D.[2,3]*

[1]Department of Psychology and Human Development, Vanderbilt University, Nashville, TN 37240, USA

[2]Center of Excellence for Stress and Mental Health, VA San Diego Healthcare System, San Diego, CA 92161 USA

[3]Department of Psychiatry, University of California San Diego, San Diego, CA 92093 USA

Table of Contents

1. **Supplementary Table 1……………………………………………………………………………………………………3**
    a. **a.** Lists the top 600 NeuroSynth terms and lists decisions made by authors for term inclusion
2. **Supplementary Table 2……………………………………………………………………………………………………14**
    a. Lists the 60 NeuroSynth terms that were cross-checked with PubMed searches using the NeuroSynth term and PTSD or PTSD+fMRI
3. **Supplementary Table 3……………………………………………………………………………………………………16**
    a. Lists the 41 NeuroSynth terms used in graph metric and clustering analyses
4. **Supplementary Table 4……………………………………………………………………………………………………17**
    a. Cluster stability estimates from 500 bootstrap iterations at the selected 15% threshold
5. **Supplementary Figure 1…………………………………………………………………………………………………18**
    a. Flow diagram detailing the protocol of identifying and selecting Neurosynth terms for inclusion in the present study.
6. **Supplementary Figure 2…………………………………………………………………………………………………19**
    a. Representation of PTSD study coordinates within Neurosynth resting-state maps.
7. **Supplementary Figure 3…. ………………………………………………………………………………………………20**
    a. Representation of PTSD study coordinates within CAREN resting-state maps.
8. **Supplementary Figure 4…………………………………………………………………………………………………21**
    a. Conceptual framework integrated with clustering results.
9. **Supplementary Figure 5…………………………………………………………………………………………………22**
    a. Distribution of contributing studies across Neurosynth terms
10. **Supplementary Figure 6…………………………………………………………………………………………………23**
    a. Cumulative frequency distribution of PTSD-related and PTSD+fMRI PubMed term counts
11. **Supplementary Figure 7…………………………………………………………………………………………………24**
    a. Threshold evaluation for selecting the top-correlation cutoff used to construct the Concept Brain Network maps
12. **Supplementary Figure 8…………………………………………………………………………………………………25**
    a. Threshold stability across candidate clustering thresholds
13. **Supplementary Figure 9…………………………………………………………………………………………………26**
    a. Cluster assignment stability across candidate graph thresholds.
14. **Supplementary Figure 10………………………………………………………………………………………………27**
    a. Binarized heatmap of fast greedy community detection
15. **Supplementary Figure 11………………………………………………………………………………………………28**
    a. Binarized graph network plot
16. **Supplementary Figure 12………………………………………………………………………………………………29**
    a. Evaluation of ROI radius for constructing PTSD study-specific overlap maps

Supplementary Table 1. NeuroSynth terms and decisions for inclusion in analyses

NeuroSynth Term	# studies	# voxels	Type-Final	PTSD-Final	Downloaded
abilities	291	11638	construct		
ability	1094	38134	construct*		Y
abstract	289	11245	other		
acc	558	19397	brain		
accumbens	200	6435	brain		
accuracy	632	20629	construct		
accurate	254	10341	other		
acoustic	171	5598	other		
action	886	34656	construct		Y
actions	634	24272	construct		
ad	196	5187	other		
adaptation	308	9594	other*		
addition	1399	49750	other		
adolescence	241	7369	other		
adolescent	186	4690	other		
adolescents	455	13665	other		
adult	486	16706	other		
adulthood	172	6014	construct*		
adults	1406	51685	other		
affect	625	22223	construct	PTSD	
affective	748	28542	construct	PTSD	Y
age	1870	62849	construct		Y
aging	293	12148	other*		
al	276	10043	other		
alzheimer	267	8375	construct		
alzheimer disease	263	8164	construct		
amygdala	1579	54873	brain		
angular	389	12936	brain		
angular gyrus	310	10782	brain		
animal	246	8576	other		
anterior	3932	146983	brain		
anterior cingulate	2086	73375	brain		
anterior insula	681	28520	brain		
anterior posterior	245	9128	other		
anterior temporal	194	6488	brain		
anticipation	301	10474	construct	PTSD	
anxiety	449	13053	construct	PTSD	
approach	943	30472	other*		
arousal	295	11556	construct	PTSD	
association	880	27953	other		
associations	578	18448	other		
associative	295	11344	other		
atrophy	206	6472	other		
Cognition	1831	65346	construct	PTSD	Y
Cognitional	824	29856	construct	PTSD	Y
auditory	1252	46557	other*		
auditory cortex	279	8290	brain		
autism	244	11329	construct		
autism spectrum	170	8237	construct		
aversive	238	8529	construct	PTSD	
awareness	290	9504	construct		
ba	399	13839	other*		
basal	552	24130	other		

Supplementary Table 1 Continued.

basal ganglia	483	21757	brain		
baseline	600	21739	other		
behavior	1204	41654	other*		
behavioral	2311	79961	other*		
behavioral performance	238	8163	construct		
behaviors	335	12281	other*		
bias	311	9517	other		
bilaterally	685	25967	brain*		
blind	288	10456	other		
body	552	19337	other*		
brainstem	171	7026	brain		
broca	223	8208	brain		
brodmann	239	8402	brain		
capacity	298	11644	construct*		
categories	284	9348	other		
category	353	12135	other		
caudate	718	25648	brain		
caudate nucleus	277	11090	brain		
cerebellar	498	22415	brain		
cerebellum	1052	47414	brain		
cerebral	911	31163	brain		
childhood	233	6847	other		
children	621	21386	other		
chinese	214	8314	other		
choice	326	11334	construct*		
choices	194	6474	other		
chronic	399	12103	construct		
cingulate	3361	119720	brain		
cingulate cortex	1938	66124	brain		
cingulate gyrus	350	11779	brain		
classical	170	6587	other		
cognition	731	25308	construct	PTSD	Y
cognitive	3427	120005	construct	PTSD	Y
cognitive control	598	19614	construct	PTSD	
cognitive functions	188	6715	construct	PTSD	
cognitive impairment	232	7063	construct	PTSD	
cognitive performance	174	5247	construct	PTSD	
cognitive processes	302	10686	construct	PTSD	
color	236	8092	other		
communication	271	9047	other		
compensatory	327	13613	construct		
complex	1135	43000	other		
complexity	225	10187	other		
comprehension	424	15365	construct		
conceptual	192	6275	construct		
concurrent	174	6125	other		
conflict	337	10925	construct		
congruent	267	9876	construct		
conjunction	306	12628	other		
connectivity	2636	94335	other		
conscious	182	6689	construct		
context	986	35713	construct	PTSD	Y
contexts	223	8606	brain*		
contextual	220	8593	construct	PTSD	
contralateral	448	18149	brain*		

Supplementary Table 1 Continued.

control	3796	137024	construct	PTSD	Y
control task	172	7222	other		
controlled	516	19406	other*		
controlling	308	9658	other		
controls	2411	76397	other		
core	477	19760	other		
correct	359	12975	other		
cortex acc	410	13764	brain		
cortex anterior	350	14475	brain		
cortex dlpfc	383	12422	brain		
cortex inferior	256	9679	brain		
cortex mpfc	263	7870	brain		
cortex ofc	183	4892	brain		
cortex pfc	278	10295	brain		
cortex posterior	223	8752	brain		
cortex superior	179	7618	brain		
cortical	2677	98339	brain		
cortices	1470	61456	brain		
cortico	179	8725	other		
cue	452	16837	construct*		
cued	250	9605	other*		
cues	762	27689	other		
cuneus	201	7220	brain		
damage	255	7769	construct		
decision	800	28630	construct	PTSD	Y
decision making	509	16413	construct	PTSD	
decisions	375	12239	construct	PTSD	
decline	211	7469	other		
default	907	31297	other		
default mode	777	26256	brain		
deficit	432	15214	construct	PTSD	
deficits	972	33780	construct*	PTSD*	Y
delay	359	12214	other		
delayed	274	8989	other		
demand	186	7441	other		
demanding	180	6728	other		
demands	596	24759	construct*		
depressed	214	4882	construct	PTSD	
depression	502	11677	construct	PTSD	
depressive	337	7501	construct	PTSD	
depressive disorder	218	5154	construct	PTSD	
detect	308	10270	other		
detected	410	14756	other		
detection	604	22260	other		
development	736	22753	other*		
developmental	400	14466	other*		
diagnosis	203	4895	other		
difficult	328	11764	other		
difficulty	410	15968	construct		
dimensions	199	7338	other		
discrimination	346	12705	other		
disease	766	24827	other		
disorder	1358	39380	construct	PTSD	Y
disorders	899	30902	construct*	PTSD*	Y
divided	175	6956	other		

Supplementary Table 1 Continued.

dlpfc	489	16139	brain		
dmn	366	9650	brain		
dominant	227	10356	other		
dopamine	255	7822	construct		
dopaminergic	205	7013	construct		
dorsal	1727	62764	brain		
dorsal anterior	410	12673	brain		
dorsal premotor	193	8767	brain		
dorsal ventral	175	7574	brain		
dorsolateral	1286	45731	brain		
dorsolateral prefrontal	1049	36216	brain		
dorsomedial	279	9566	brain		
dorsomedial prefrontal	178	5630	brain		
drug	319	8459	construct		
education	193	4571	other		
efficiency	244	7658	other		
effort	204	7905	brain*		
emotion regulation	247	8531	construct	PTSD	
emotional	1708	58326	construct	PTSD	Y
emotional stimuli	212	6776	construct	PTSD	
emotionally	214	7318	construct	PTSD	
emotions	444	15877	construct	PTSD	
empathy	187	7913	construct		
encoded	255	9424	other		
encoding	882	34775	construct	PTSD	Y
engagement	349	15754	other*		
episode	190	4440	other		
episodic	488	20164	construct		
episodic memory	332	13518	construct*	PTSD*	
error	464	16093	construct		
errors	347	12771	other*		
evaluation	394	13373	other		
events	636	25283	other		
everyday	223	7626	other		
execution	395	19626	other		
executive	786	28937	construct	PTSD	Y
executive control	230	8253	construct	PTSD	
experience	759	27779	construct*	PTSD*	Y
experiences	267	9909	other		
explicit	302	11474	construct		
expression	328	10688	construct*		
expressions	362	12989	construct		
extrastriate	246	8997	brain		
eye	417	17591	other*		
face	896	31842	other		
faces	864	29833	other		
facial	502	17104	construct		
facial expressions	250	8492	construct		
familiar	275	10535	other		
familiarity	188	7551	construct		
fc	206	5655	other		
fear	363	12269	construct	PTSD	
fearful	221	6575	construct	PTSD	
feature	428	13105	other		
features	723	24904	other		

Supplementary Table 1 Continued.

female	392	12189	construct		
females	272	10397	construct*		
finger	330	17212	other*		
focus	348	11316	other*		
food	171	5071	other		
form	599	20062	other		
forms	244	10272	other		
frequency	714	21117	other		
frontal	4735	175560	brain		
frontal cortex	803	27535	brain		
frontal gyri	249	9593	brain		
frontal gyrus	1983	70487	brain		
frontal lobe	211	7007	brain		
frontal parietal	428	17030	brain		
frontal temporal	197	7563	brain		
fronto	775	29114	brain		
fronto parietal	424	16766	brain		
frontoparietal	360	13467	brain		
functional connectivity	1829	65616	other		
fusiform	972	35417	brain		
fusiform gyrus	582	21231	brain		
gain	204	8478	other		
game	176	5246	other		
ganglia	485	21859	brain		
gender	484	14257	other*		
generation	335	15216	other		
goal	582	21308	construct*		
goal directed	216	8840	construct		
gyri	729	26274	brain		
gyrus	4129	143923	brain		
gyrus ifg	309	10903	brain		
gyrus inferior	288	10837	brain		
gyrus middle	214	7112	brain		
gyrus superior	207	7915	brain		
gyrus superior	207	7915	brain		
hand	879	35364	other		
handed	368	16848	other*		
happy	225	6964	construct		
hc	245	6269	other		
head	202	6614	other		
health	170	4317	other		
hemisphere	872	32580	other*		
hemispheres	239	9445	brain*		
hemispheric	398	14635	other*		
hippocampal	566	21360	brain		
hippocampus	1059	38697	brain		
human	2400	82598	other		
humans	985	36850	other		
hyperactivity	222	6966	other		
identification	330	11505	other		
ifg	386	14157	brain		
illness	187	4283	construct		
image	327	10383	other		
imagery	312	14710	construct		
images	822	27436	other		

Supplementary Table 1 Continued.

implicit	263	9175	construct		
incongruent	266	8956	construct		
inferior	3374	126623	other		
inferior frontal	1890	72219	brain		
inferior parietal	1159	44943	brain		
inferior temporal	340	11952	brain		
information	2647	93756	other*		
inhibition	601	19800	construct	PTSD	
inhibitory	328	10931	construct		
insula	2017	78194	brain		
insular	487	17346	brain		
insular cortex	228	8853	brain		
integrated	226	7835	construct		
integrating	178	5963	brain*		
integration	771	27390	construct		Y
integrity	235	7045	other		
intensity	428	15937	other		
interaction	1270	43463	other		
interactions	833	31544	other		
interference	330	13013	construct	PTSD	
interval	182	6771	other		
intraparietal	671	26519	brain		
intraparietal sulcus	571	22614	brain		
ips	212	7574	other		
ipsilateral	236	9610	brain*		
item	269	9996	other		
items	448	15962	other		
judgment	290	10373	construct*	PTSD*	
judgments	372	12949	construct	PTSD	
junction	510	18906	other*		
knowledge	635	23228	construct		
language	1101	42749	construct		Y
lateral	1553	59614	brain*		
lateral occipital	226	7125	brain		
lateral prefrontal	359	12382	brain		
lateralization	205	7412	brain*		
lateralized	349	14459	brain*		
learned	361	14676	other		
learning	1144	43622	construct	PTSD	Y
lesion	282	9098	other*		
lesions	236	8226	brain*		
letter	173	6818	other		
lexical	331	14271	construct		
life	488	16446	other		
light	302	10259	other		
limbic	502	18957	brain		
lingual	263	8998	other		
lingual gyrus	175	5329	brain		
linguistic	315	14049	construct		
listening	250	9819	construct		
load	366	15166	other*		
lobe	1250	47290	brain		
lobes	370	14031	brain		
lobule	833	31588	brain		
location	499	18262	other		

Supplementary Table 1 Continued.

locations	253	8615	other		
long term	335	11769	other		
loss	396	12393	construct	PTSD	
magnitude	464	15416	other		
maintenance	350	14313	construct		
major depressive	224	5251	construct	PTSD	
manipulation	310	12333	other		
mapping	520	17813	other		
maps	396	13540	other		
matching	295	11856	other		
mdd	185	4001	construct	PTSD	
medial	2674	100331	brain		
medial frontal	525	19417	brain		
medial prefrontal	983	34109	brain		
medial temporal	456	20099	brain		
memories	256	10113	construct	PTSD	
memory	2744	101915	construct	PTSD	Y
memory performance	255	8433	construct	PTSD	
memory retrieval	228	9305	construct	PTSD	
memory task	383	12535	construct	PTSD	
memory wm	259	10740	construct	PTSD	
midbrain	199	8182	brain		
middle frontal	682	24148	brain		
middle occipital	176	6258	brain		
middle temporal	729	25303	brain		
midline	186	6788	brain		
mind	313	12342	other		
mirror	240	10618	other		
modalities	299	12245	other		
modality	380	16369	other		
monetary	300	9318	construct		
monitoring	465	16686	other*		
mood	238	6075	construct	PTSD	
motion	451	16050	construct		
motivation	189	6977	construct		
motivational	192	6679	construct		
motor	2565	109491	construct		Y
motor control	214	8683	construct		
motor cortex	438	19990	brain		
motor sma	191	7710	brain		
movement	670	28998	construct		Y
movements	674	32758	construct		Y
mpfc	323	10162	brain		
ms	321	9787	construct		
naming	179	7361	other		
natural	221	7072	other		
nature	435	15419	other		
negative	1567	50373	other		
network dmn	332	8644	brain		
neutral	922	31948	other		
noise	267	8753	other		
novel	1089	39798	other		
nucleus	644	24205	brain		
nucleus accumbens	194	6217	brain		
object	851	29742	other		

Supplementary Table 1 Continued.

objects	692	25666	other		
occipital	1470	52026	brain		
occipital cortex	342	11674	brain		
occipital gyrus	239	7875	brain		
occipito	207	8111	brain		
occipitotemporal	176	6403	brain		
ofc	227	7221	brain		
older adults	310	12991	other		
operculum	229	9776	brain		
orbitofrontal	851	28814	brain		
orbitofrontal cortex	665	22442	brain		
organization	458	18135	other		
orientation	184	6019	other		
outcome	385	14673	other		
outcomes	372	11574	other		
pain	516	23295	construct		
painful	189	8214	construct		
paired	184	6729	other		
pairs	346	13036	other		
parahippocampal	602	22715	brain		
parahippocampal gyrus	327	12887	brain		
parietal	4132	163276	brain		
parietal cortex	1076	41180	brain		
parietal cortices	239	9972	brain		
parietal junction	198	7838	brain		
parietal lobe	368	14789	brain		
parietal lobule	731	26445	brain		
parietal network	206	7673	brain		
parieto	192	7182	brain		
parkinson	179	6763	construct		
parkinson disease	175	6621	construct		
passive	303	12522	construct		
pcc	209	6509	brain		
people	693	24285	other		
perception	1278	45576	construct		Y
perceptual	867	28917	construct		Y
performance	2714	97988	construct		Y
person	358	15169	other		
personal	191	6802	construct*	PTSD*	
personality	309	9843	construct		
pfc	521	20550	brain		
phonological	377	17844	construct		
picture	353	12970	other		
pictures	554	20764	other		
place	189	6595	other		
placebo	332	10412	construct		
planning	258	10304	construct*		
plasticity	215	7358	brain*		
pole	249	8812	other		
poor	222	7864	other		
positive negative	308	10863	construct		
postcentral	290	10050	brain		
postcentral gyrus	184	6004	brain		
posterior	3208	123740	brain*		
posterior cingulate	947	32721	brain		

Supplementary Table 1 Continued.

posterior insula	175	7565	brain		
posterior parietal	476	18933	brain		
posterior superior	343	14515	brain		
precentral gyrus	255	8533	brain		
precuneus	1014	36712	brain		
prediction	365	12732	other		
predictions	211	7671	other		
predictive	316	11866	construct		
prefrontal	4400	157801	brain		
prefrontal cortex	3254	112230	brain		
prefrontal cortices	229	9651	brain		
premotor	1036	48163	brain		
premotor cortex	600	27010	brain		
primary	1271	49076	other		
primary motor	321	16209	brain		
primary somatosensory	191	7549	brain		
priming	202	5235	construct		
probability	171	5574	other		
production	318	13945	other		
putamen	599	24105	brain		
ratings	418	14727	other		
reaction	551	18950	construct		
reaction time	298	10575	construct		
reaction times	214	7227	construct		
reactivity	257	7286	construct	PTSD	
read	201	8389	other		
reading	521	21842	construct		
reasoning	182	6641	construct		
recall	244	10992	construct		
recognition	1001	35156	construct		Y
reference	201	6804	other		
referential	176	5038	other		
regulation	598	20125	construct	PTSD	
relevance	286	9734	other		
reorganization	194	8554	other		
repetition	262	8950	construct*		
response inhibition	218	7438	construct	PTSD	
rest	482	18834	other		
resting	1565	44595	other		
resting state	1421	38975	other		
retrieval	872	36621	construct	PTSD	Y
reward	922	30418	construct	PTSD	Y
rewards	246	7562	construct	PTSD	
risk	708	18379	construct		Y
rostral	376	14831	brain		
rs	212	5498	other		
rule	172	6537	other		
salience	396	12228	other*		
salient	257	8038	other*		
schizophrenia	715	20121	construct		Y
selection	483	18586	other		
selective	723	24080	other		
self reported	184	5982	other		
semantic	1031	40030	construct		Y
sensitivity	667	21956	other		

Supplementary Table 1 Continued.

sensorimotor	684	29847	brain		
sensorimotor cortex	172	8404	brain		
sensory	1145	43001	other*		
sensory motor	181	7581	brain*		
sentence	307	11204	construct*		
sentences	356	13461	construct		
sequence	379	15766	other		
sequences	288	11811	other		
sex	391	12817	construct		
short term	199	7654	other		
similarity	196	6549	other		
size	336	10220	other		
social	1302	47083	construct	PTSD	Y
social cognition	220	8247	construct		
somatosensory	674	28760	other*		
somatosensory cortex	316	12214	brain		
sound	271	9667	other		
sounds	278	9307	other*		
source	408	13349	other		
sources	234	8093	other		
space	303	10114	other		
spatial	1424	53646	construct		Y
spatially	259	8658	other		
spectrum	269	11824	other		
speech	642	23574	construct		Y
spoken	186	7023	other		
spontaneous	361	9205	other		
stimulation	909	32733	construct		Y
strategies	357	13597	other		
strategy	253	9019	other		
stream	247	8715	other		
stress	321	8294	construct	PTSD	
striatal	545	21039	brain		
striatum	886	31019	brain		
stroop	225	7134	construct		
sts	203	6456	other		
subcortical	648	27192	brain		
subjective	458	16789	other*		
subsequent	704	24847	other		
success	184	7043	other		
sulcus	1272	48947	brain		
sulcus ips	173	6399	brain		
superior	2839	105064	other		
superior parietal	622	24152	brain		
superior temporal	1422	50978	brain		
supplementary	827	35336	other		
supplementary motor	748	31848	brain		
suppression	314	11814	construct	PTSD	
supramarginal	375	13835	brain		
supramarginal gyrus	296	11421	brain		
sustained	313	12408	other		
switching	193	6510	brain*		
symptom	338	9534	construct*		
symptoms	660	18172	construct		Y
tactile	225	8648	construct		

Supplementary Table 1 Continued.

target	853	31187	other		
targets	349	11689	other		
task	5993	223010	other		
task relevant	177	5853	other		
tasks	2228	96772	other		
temporal	4526	169500	other*		
temporal cortex	570	20134	brain		
temporal gyri	220	7409	brain		
temporal gyrus	1138	39137	brain		
temporal lobe	643	24983	brain		
temporal lobes	183	7661	brain		
temporal parietal	216	9108	brain		
temporal sulcus	518	18785	brain		
temporo	354	14804	brain		
temporo parietal	268	11585	brain		
temporoparietal	262	9247	brain		
temporoparietal junction	178	6295	brain		
thalamic	235	8295	brain		
thalamus	922	35226	brain		
theory	522	19771	construct		
theory mind	181	7761	construct		
thought	573	20879	construct	PTSD	
timing	186	7094	other		
trait	360	9778	construct		
traits	227	7423	construct		
unfamiliar	183	7406	construct*		
valence	361	13519	other*		
value	470	15226	other		
values	415	10742	other		
ventral	1729	63054	brain		
ventral premotor	185	8783	brain		
ventral striatum	415	12989	brain		
ventrolateral	463	18976	brain		
ventrolateral prefrontal	314	11411	brain		
ventromedial	437	15051	brain		
ventromedial prefrontal	333	12062	brain		
verbal	729	26713	other		
video	190	8448	other		
viewing	463	16803	construct		
visual	3110	115726	other		
visual cortex	488	16214	brain		
visual stimuli	270	8526	other		
visually	333	14885	other*		
visuospatial	267	10517	other*		
vmpfc	199	6971	brain		
vs	865	31899	other		
vulnerability	188	5212	construct	PTSD	
wm	339	13110	other*		
word	944	36501	other		
words	948	38353	other		
working	1126	41359	other*		
working memory	1091	39905	construct	PTSD	Y

Note. *=decision made by majority (2/3 of raters agreed)

Supplementary Table 2. Top 69 NeuroSynth terms used to identify PTSD relevant terms

NeuroSynth term	# studies	# voxels	max r	median r	sizes	mean s	max s	Pubmed	PubMesh	PubmedF	PubmeshF	Drop
affect	625	22223	0.458	0.005	478	4.98	7.75	6612	490	257	21	
affective	748	28542	0.620	0.016	5015	5.04	14.39	1568	0	122	0	
anticipation	301	10474	0.736	0.008	4023	6.15	17.69	279	0	29	0	
anxiety	449	13053	0.707	0.005	1647	5.58	9.95	13424	3534	337	53	
arousal	295	11556	0.547	0.009	2696	4.97	11.18	2107	1048	138	82	
Cognition	1831	65346	0.512	0.000	3819	4.59	8.32	2996	425	175	42	
Cognitional	824	29856	0.512	0.000	3183	4.66	8.87	2996	0	175	0	RD
aversive	238	8529	0.569	0.022	3619	5.05	12.11	488	0	36	0	
cognition	731	25308	0.166	0.000	464	4.79	7.30	8619	1645	482	67	
cognitive	3427	120005	0.213	0.002	963	4.62	6.81	8619	0	482	0	
cognitive control	598	19614	0.190	0.001	1420	4.69	8.18	2288	6	274	0	
cognitive functions	188	6715	0.005	0.000	353	5.03	7.79	3113	1645	220	67	ND
cognitive impairment	232	7063	0.120	0.003	1352	4.83	9.55	963	153	62	10	
cognitive performance	174	5247	0.037	0.000	102	5.42	9.03	1178	0	125	0	ND
cognitive processes	302	10686	0.070	0.000	136	4.94	6.83	2122	0	220	0	ND
context	986	35713	0.000	0.000	4	5.42	5.51	3193	0	106	0	ND
contextual	220	8593	0.083	0.002	787	4.63	6.75	820	0	43	0	
control	3796	137024	0.123	0.000	932	4.62	7.03	10480	20	857	0	
decision	800	28630	0.656	0.004	1480	4.89	9.13	977	0	29	0	
decision making	509	16413	0.656	0.004	1680	4.91	9.37	831	567	28	16	RD
decisions	375	12239	0.383	0.002	626	4.77	6.89	977	0	29	0	RD
deficit	432	15214	0.056	0.000	88	5.33	8.43	1616	0	160	0	ND
deficits	972	33780	0.051	0.000	52	5.24	6.38	1616	0	160	0	ND
depressed	214	4882	0.087	0.000	21	5.33	6.05	16602	0	338	0	ND
depression	502	11677	0.177	0.000	148	4.95	9.09	16602	7857	338	115	
depressive	337	7501	0.361	0.000	27	5.40	6.45	16602	0	338	0	ND
depressive disorder	218	5154	0.914	0.000	113	5.34	8.06	6005	3479	137	79	
disorder	1358	39380	0.150	0.000	251	4.35	5.60	47164	154	1577	0	
disorders	899	30902	0.396	0.005	351	4.99	7.63	47164	154	1577	0	RD
emotion regulation	247	8531	0.520	0.010	1810	4.90	9.19	1011	141	140	7	RD
emotional	1708	58326	0.707	0.015	8428	5.69	22.80	14742	10736	608	403	
emotional stimuli	212	6776	0.690	0.004	1397	5.48	12.72	608	0	153	0	RD
emotionally	214	7318	0.640	0.007	2374	5.22	10.56	426	0	29	0	RD
emotions	444	15877	0.647	0.020	2922	5.25	11.95	14742	10736	608	403	RD
encoding	882	34775	0.487	0.011	8414	4.85	10.63	322	0	60	0	
episodic memory	332	13518	0.514	0.009	10987	4.92	13.27	265	173	34	13	
executive	786	28937	0.273	0.003	2302	4.49	7.81	783	0	127	0	
executive control	230	8253	0.140	0.001	352	4.88	8.12	431	182	85	28	
experience	759	27779	0.033	0.000	87	5.03	6.45	10035	0	199	0	ND
fear	363	12269	0.753	0.012	3934	6.16	16.21	3697	1819	266	138	
fearful	221	6575	0.753	0.007	2550	6.78	15.76	3908	1819	288	138	RD
inhibition	601	19800	0.519	0.001	935	4.98	8.28	965	128	75	20	
interference	330	13013	0.298	0.001	2013	4.66	7.94	567	0	31	0	
judgment	290	10373	0.210	0.000	1493	4.78	8.22	107	20	3	1	PD
judgments	372	12949	0.104	0.000	1081	4.59	6.82	107	20	3	1	PD

Supplementary Table 2 Continued.

learning	1144	43622	0.383	0.003	1598	4.67	7.78	4948	3431	398	275	
loss	396	12393	0.470	0.001	775	5.00	7.44	1860	0	62	0	
major depressive	224	5251	0.914	0.000	98	5.33	7.95	4099	0	122	0	RD
mdd	185	4001	0.615	0.000	98	5.46	8.76	876	0	50	0	ND
memories	256	10113	0.457	0.009	10956	4.65	10.81	4409	0	350	0	
memory	2744	101915	0.607	0.020	19804	4.84	12.35	4409	1934	350	149	
memory performance	255	8433	0.231	0.004	1054	4.81	7.82	817	0	110	0	
memory retrieval	228	9305	0.550	0.016	8433	4.65	11.13	432	0	45	0	RD
memory task	383	12535	0.230	0.000	447	4.81	7.18	440	0	72	0	
memory wm	259	10740	0.657	0.002	5094	4.81	11.53	40	0	9	0	PD
mood	238	6075	0.177	0.002	146	5.15	8.23	5388	490	260	21	
personal	191	6802	0.215	0.002	2912	4.59	8.50	35014	0	1149	0	
reactivity	257	7286	0.681	0.012	1516	5.79	10.77	1240	0	101	0	
regulation	598	20125	0.520	0.008	1996	4.95	9.27	3812	1162	216	2	
response inhibition	218	7438	0.519	0.002	2797	4.83	9.15	437	64	41	13	
retrieval	872	36621	0.607	0.021	21189	4.69	12.13	626	0	48	0	
reward	922	30418	0.761	0.008	12031	6.25	29.04	284	100	50	30	
rewards	246	7562	0.761	0.002	3047	6.10	15.63	284	100	50	30	RD
social	1302	47083	0.348	0.017	13049	4.75	10.55	11920	0	179	0	
stress	321	8294	0.163	0.000	185	5.03	7.38	46508	0	1542	0	
suppression	314	11814	0.056	0.000	96	5.31	6.69	623	0	35	0	ND
thought	573	20879	0.027	0.000	265	4.92	6.94	2415	810	99	22	ND
vulnerability	188	5212	0.070	0.000	6	5.87	8.69	2738	0	104	0	ND
working memory	1091	39905	0.657	0.000	11710	5.32	12.50	469	166	55	28	

Note. Pubmed column = the PubMed search term using the format: "(\"stress disorders, post traumatic\"[MeSH Terms] OR (\"stress\"[All Fields] AND \disorders\ [All Fields] AND \"post traumatic\"[All Fields]) OR \"post-traumatic stress disorders\"[All Fields] OR \"ptsd\"[All Fields]) AND 2000/01/01:2023/06/01[Date - Publication]"
PubmedF column="(\"magnetic resonance imaging\"[MeSH Terms] OR (\"magnetic\"[All Fields] AND \"resonance\"[All Fields] AND \"imaging\"[All Fields]) OR \"magnetic imaging\"[All Fields] OR \"fmri\"[All Fields]) AND (\"stress disorders, post traumatic\"[MeSH Terms] OR (\"stress\"[All Fields] AND \"disorders\"[All Fields] AND \"post trau [All Fields]) OR \post-traumatic stress disorders\"[All Fields] OR \"ptsd\"[All Fields]) AND 2000/01/01:2023/06/01[Date - Publication]"
ND=Neurosynth Drop based on if 2 of 3 apply: 1) 1) if a NeurSynth map against other term maps max r <.1; 2) if median r of a NeuroSynth map against other term maps ==0; 3) if voxel count <100
PD=PubMed Drop based on if: 1) below 200 in pubmed with PTSD papers; 2) below 25 in pubmed PTSD and fMRI papers
RD= Redundancy drop rules were the following: contains same word and then satisfy either: (a) if *r* >.5 correlation with other term, or (b) concepts with added "(s)". When redundant concepts were identified, we chose the term that had the most NeuroSynth studies contributing.

Supplementary Table 3. 41 PTSD-associated NeuroSynth terms and graph metric statistics

NeuroSynth Terms	Degree binary-top 15%	Page Rank	ClustFast Greedy	Walktrap Cluster	Betweenness	Page Rank	# studies	sizes	Pubmed
affect	12	0.037	1	Emotion	1.79	0.037	748	5015	1568
affective	10	0.031	1	Emotion	105.62	0.031	625	478	6612
anticipation	5	0.021	3	Decision Making	10.46	0.021	301	4023	279
anxiety	11	0.034	1	Emotion	53.70	0.034	449	1647	13424
arousal	11	0.034	1	Emotion	12.14	0.034	295	2696	2107
attention	2	0.013	4	Cognition	0.00	0.013	1831	3819	2996
aversive	12	0.038	1	Emotion	62.54	0.038	238	3619	488
cognition	1	0.007	1	Emotion	0.00	0.007	731	464	8619
cognitive	4	0.022	4	Cognition	1.33	0.022	3427	963	8619
cognitiveControl	4	0.021	4	Cognition	0.67	0.021	598	1420	2288
cognitiveImpairment	0	0.004	6	Independent	0.00	0.004	232	1352	963
contextual	0	0.004	7	Independent	0.00	0.004	220	787	820
control	0	0.004	8	Independent	0.00	0.004	3796	932	10480
decision	4	0.018	3	Decision Making	0.00	0.018	800	1480	977
depression	11	0.040	1	Emotion	134.00	0.040	502	148	16602
depressiveDisorder	1	0.007	1	Emotion	0.00	0.007	218	113	6005
disorder	1	0.007	1	Emotion	0.00	0.007	1358	251	47164
emotional	13	0.043	1	Emotion	173.62	0.043	1708	8428	14742
encoding	5	0.022	2	Memory Function	0.00	0.022	882	8414	322
episodicMemory	6	0.025	2	Memory Function	5.00	0.025	332	10987	265
executive	4	0.024	4	Cognition	68.00	0.024	786	2302	783
executiveControl	1	0.009	4	Cognition	0.00	0.009	230	352	431
fear	13	0.041	1	Emotion	147.42	0.041	363	3934	3697
inhibition	1	0.026	5	Independent	0.00	0.026	601	935	965
interference	5	0.026	4	Cognition	19.77	0.026	330	2013	567
learning	4	0.018	3	Decision Making	0.00	0.018	1144	1598	4948
loss	4	0.018	3	Decision Making	0.00	0.018	396	775	1860
memories	11	0.039	2	Memory Function	548.97	0.039	256	10956	4409
memory	11	0.049	4	Cognition	378.68	0.049	2744	19804	4409
memoryPerformance	5	0.022	2	Memory Function	0.00	0.022	255	1054	817
memoryTask	3	0.016	4	Cognition	0.00	0.016	383	447	440
mood	1	0.007	1	Emotion	0.00	0.007	238	146	5388
personal	4	0.017	2	Memory Function	11.84	0.017	191	2912	35014
reactivity	12	0.038	1	Emotion	25.44	0.038	257	1516	1240
regulation	11	0.035	1	Emotion	10.69	0.035	598	1996	3812
responseInhibition	1	0.026	5	Independent	0.00	0.026	218	2797	437
retrieval	7	0.029	2	Memory Function	55.98	0.029	872	21189	626
reward	10	0.035	3	Decision Making	192.60	0.035	922	12031	284
social	12	0.040	1	Emotion	132.14	0.040	1302	13049	11920
stress	3	0.012	1	Emotion	0.00	0.012	321	185	46508
workingMemory	8	0.040	4	Cognition	63.57	0.040	1091	11710	469

Supplementary Table 4. Cluster Stability Metrics Using 15% Threshold

Metric	Method	Value	Low CI	High CI
**Jaccard**	edgedrop	0.901	0.716	1.000
	jitter	0.614	0.362	0.976
	subset	0.845	0.502	1.000
**ARI**	edgedrop	0.887	0.721	1.000
	jitter	0.540	0.325	0.761
	subset	0.822	0.590	1.000
**NMI**	edgedrop	0.941	0.854	1.000
	jitter	0.669	0.507	0.820
	subset	0.908	0.778	1.000
**VI**	edgedrop	0.189	0.000	0.455
	jitter	0.974	0.551	1.384
	subset	0.290	0.000	0.686

Note. Values reflect mean cluster stability estimates from 500 bootstrap iterations at the selected 15% threshold. Confidence intervals reflect the lower and upper bounds across bootstrap iterations. Stability was evaluated using three perturbation methods: edge drop, jitter, and subset resampling. Higher values indicate greater stability for Jaccard similarity, adjusted Rand index (ARI), and normalized mutual information (NMI), whereas lower values indicate greater stability for variation of information (VI).

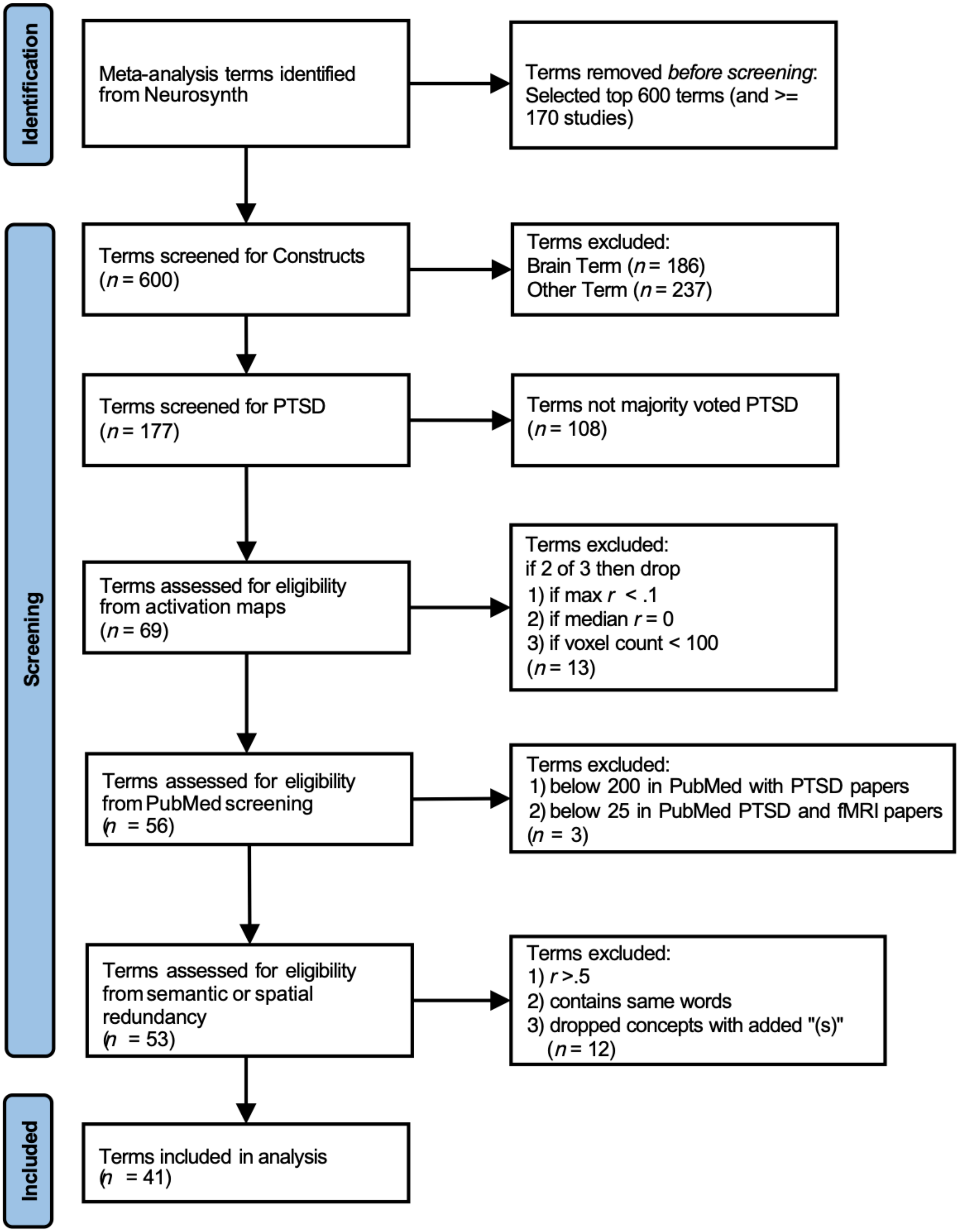

Supplementary Figure 1. Flow diagram detailing the process of identifying and selecting Neurosynth terms for inclusion in the present study.

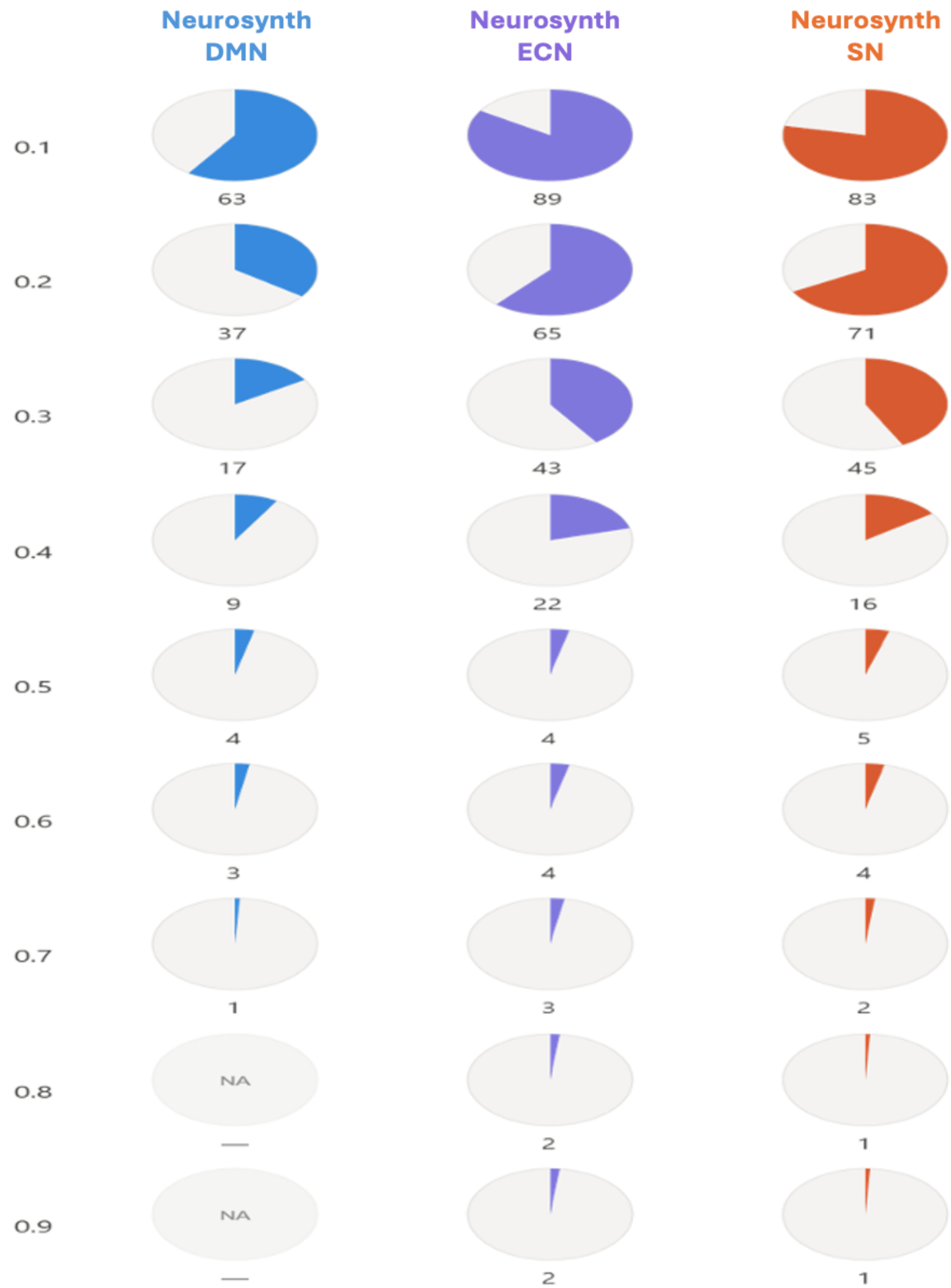

Supplementary Figure 2. Representation of PTSD study coordinates within Neurosynth resting-state maps. Spherical ROIs were created using a 6-mm radius around each reported coordinate from the 106 studies comprising the Neurosynth "PTSD" term, resulting in 106 study-specific PTSD brain maps. For each study-specific map, we quantified peak containment within each Neurosynth resting-state map, defined as the proportion of coordinate-derived ROIs that intersected the resting-state map. Higher peak-containment values indicate that a greater proportion of coordinates from a given PTSD study were represented within that resting-state map. The figure shows the number of PTSD studies meeting or exceeding each peak-containment threshold, ranging from 0.10 to 1.00. Values below each pie chart indicate the number of studies exceeding the corresponding threshold. Larger values indicate that PTSD study coordinates were more consistently represented within that resting-state map.

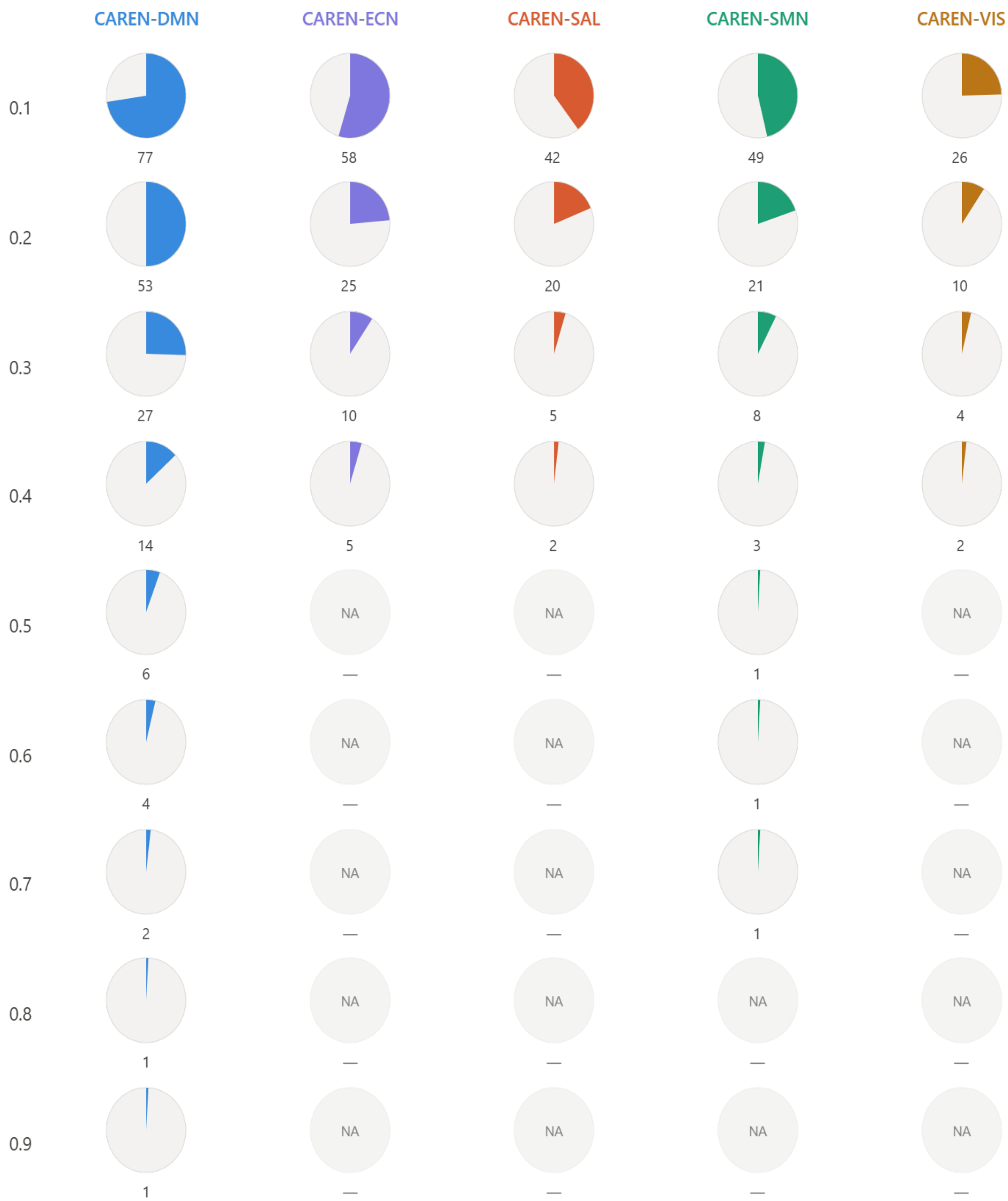

Supplementary Figure 3. Representation of PTSD study coordinates within CAREN resting-state maps. Spherical ROIs were created using a 6-mm radius around each reported coordinate from the 106 studies comprising the Neurosynth “PTSD” term, resulting in 106 study-specific PTSD brain maps. For each study-specific map, we quantified peak containment within each CAREN resting-state map, defined as the proportion of coordinate-derived ROIs that intersected the resting-state map. Higher peak-containment values indicate that a greater proportion of coordinates from a given PTSD study were represented within that CAREN resting-state map. The figure shows the number of PTSD studies meeting or exceeding each peak-containment threshold, ranging from 0.10 to 1.00. Values below each pie chart indicate the number of studies exceeding the corresponding threshold. Larger values indicate that PTSD study coordinates were more consistently represented within that CAREN resting-state map.

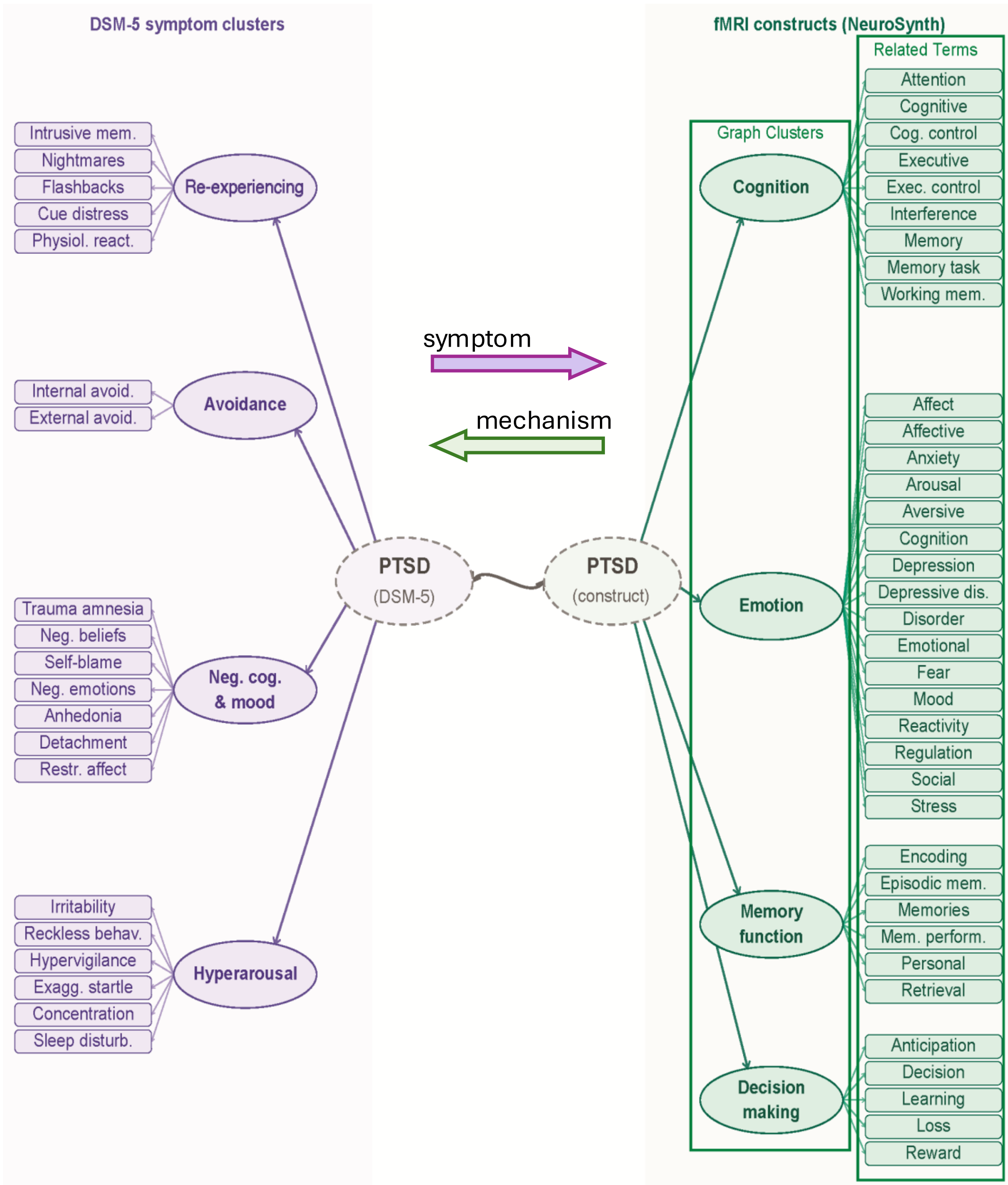

Supplementary Figure 4. Mapping selected Neurosynth terms onto the PTSD construct framework. This figure extends the conceptual model shown in Figure 1 by displaying the specific Neurosynth terms retained for analysis. The left side depicts PTSD as a latent DSM-5 clinical construct defined by symptom clusters, whereas the right side shows how PTSD-relevant fMRI terms fMRI terms were organized into graph-derived construct clusters based on spatial similarity among whole-brain Neurosynth brain maps. These concept clusters: cognition, emotion, memory function, and decision-making, represent neuroimaging-informed construct domains rather than PTSD-specific neural abnormalities.

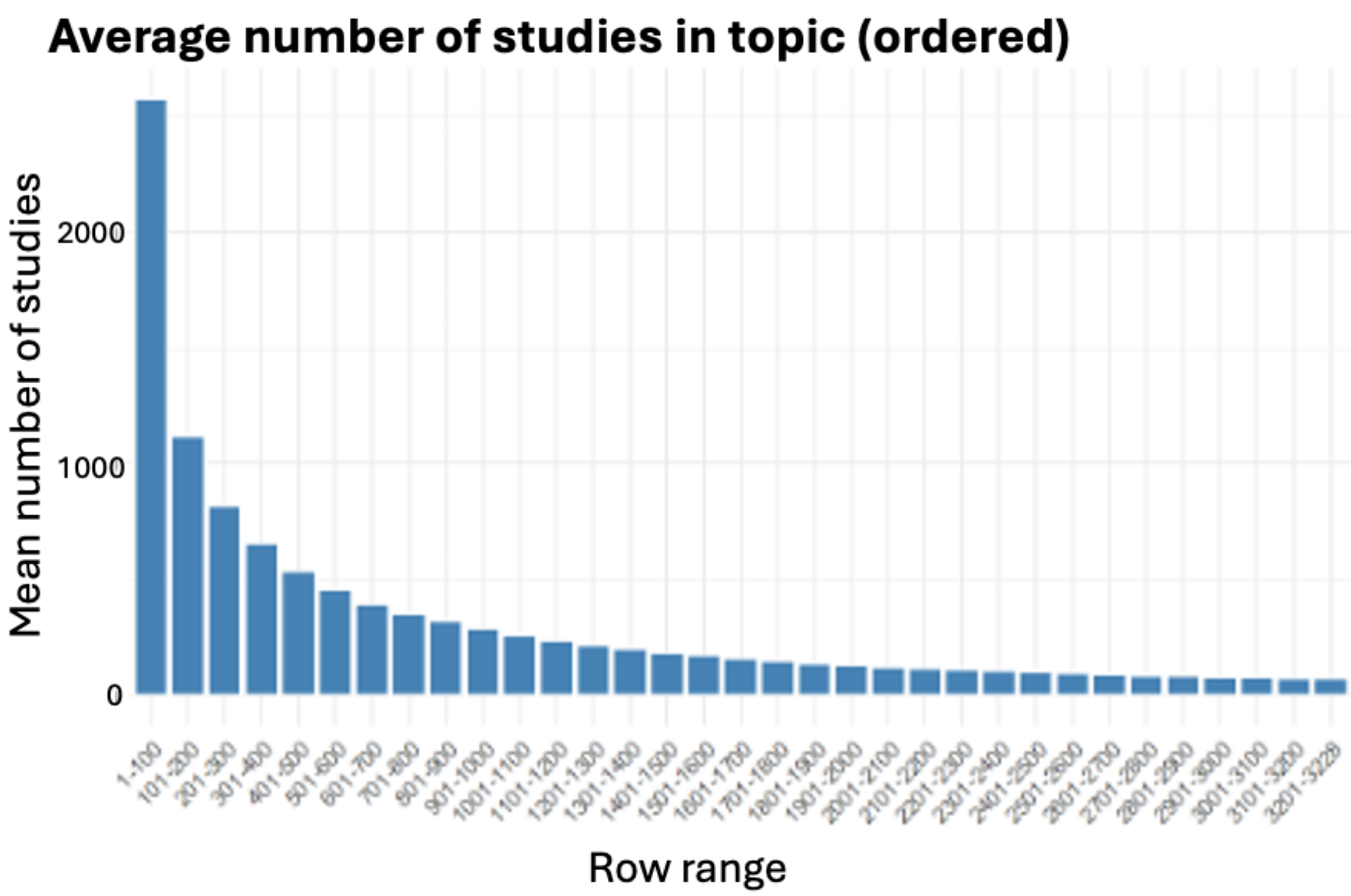

Supplementary Figure 5. Distribution of contributing studies across Neurosynth terms. Neurosynth terms were ordered by the number of contributing studies and summarized in bins of 100 terms. The distribution showed a deep decline in meta-analytic support across terms, with an elbow-based cutoff suggesting a threshold of 872 contributing studies, which would have retained 221 terms. Because this threshold was considered overly restrictive for persevering a broad psychological construct space, the top 600 Neurosynth terms were retained for subsequent construct classification and filtering.

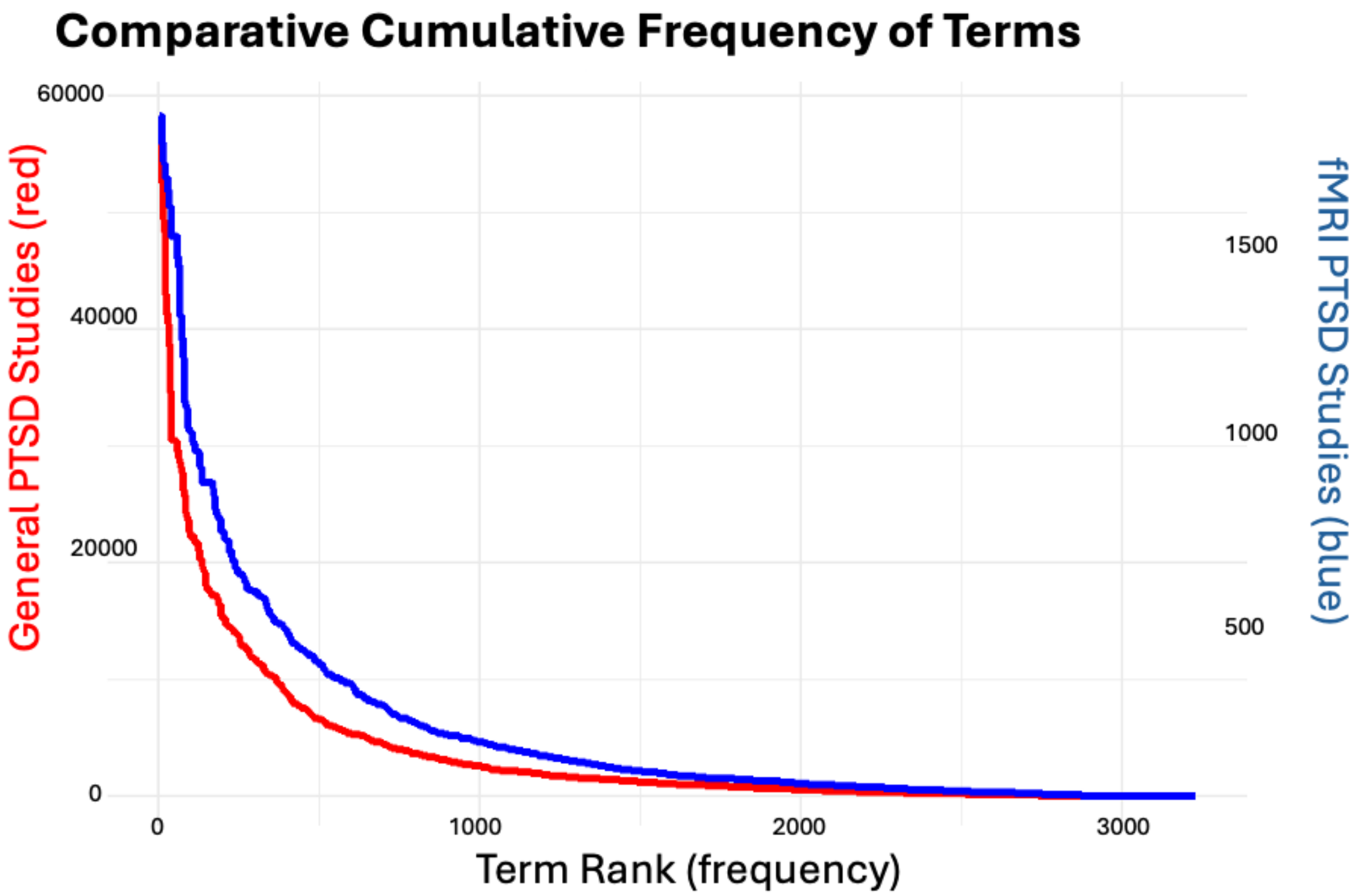

Supplementary Figure 6. Cumulative frequency distribution of PTSD-related and PTSD+fMRI PubMed term counts. Terms were rank ordered by PubMed publication frequency for searches combining each candidate Neurosynth term with “PTSD” and “PTSD+fMRI” using both “all fields” and “MeSH terms.” The red curve shows the distribution for PTSD-related publications, and the blue curve shows the distribution for PTSD+fMRI publications. Both distributions showed elbow-like patterns, with elbow-based thresholds of 485 for PTSD-related publications and 613 terms for PTSD+fMRI publications.

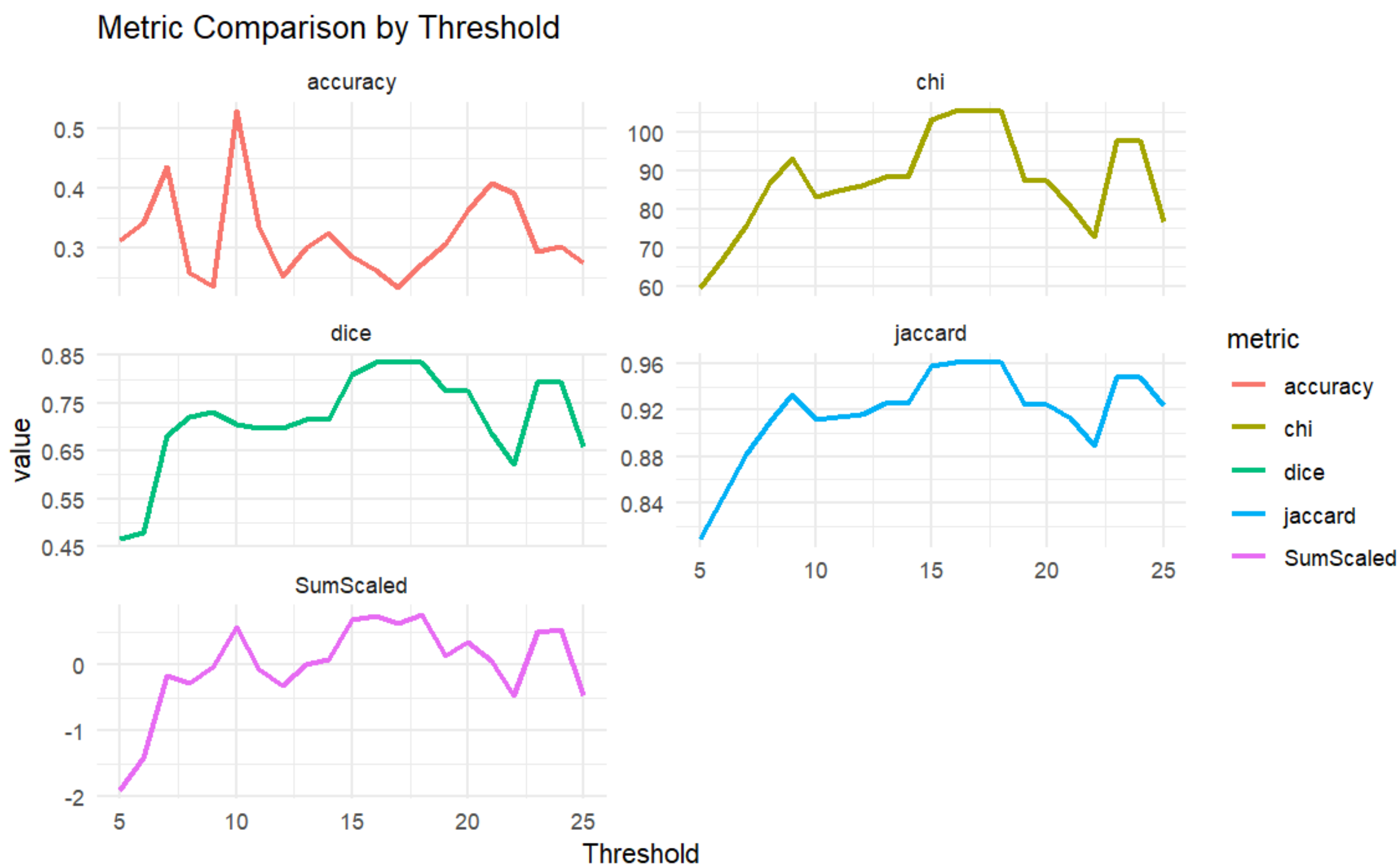

Supplementary Figure 7. Threshold evaluation for selecting the top-correlation cutoff used to construct the Concept Brain Network maps. Candidate thresholds ranging from the top 5% to 25% of unique correlations were evaluated across multiple performance metrics, including accuracy, chi-square, Dice coefficient, Jaccard index, and a summed scale metric. Metrics were compared across thresholds to identify the cutoff that provided the strongest overall balance across graph-performance indices. Based on the combined pattern of results, the top 15% correlation threshold was selected for the primary network construction and clustering analyses.

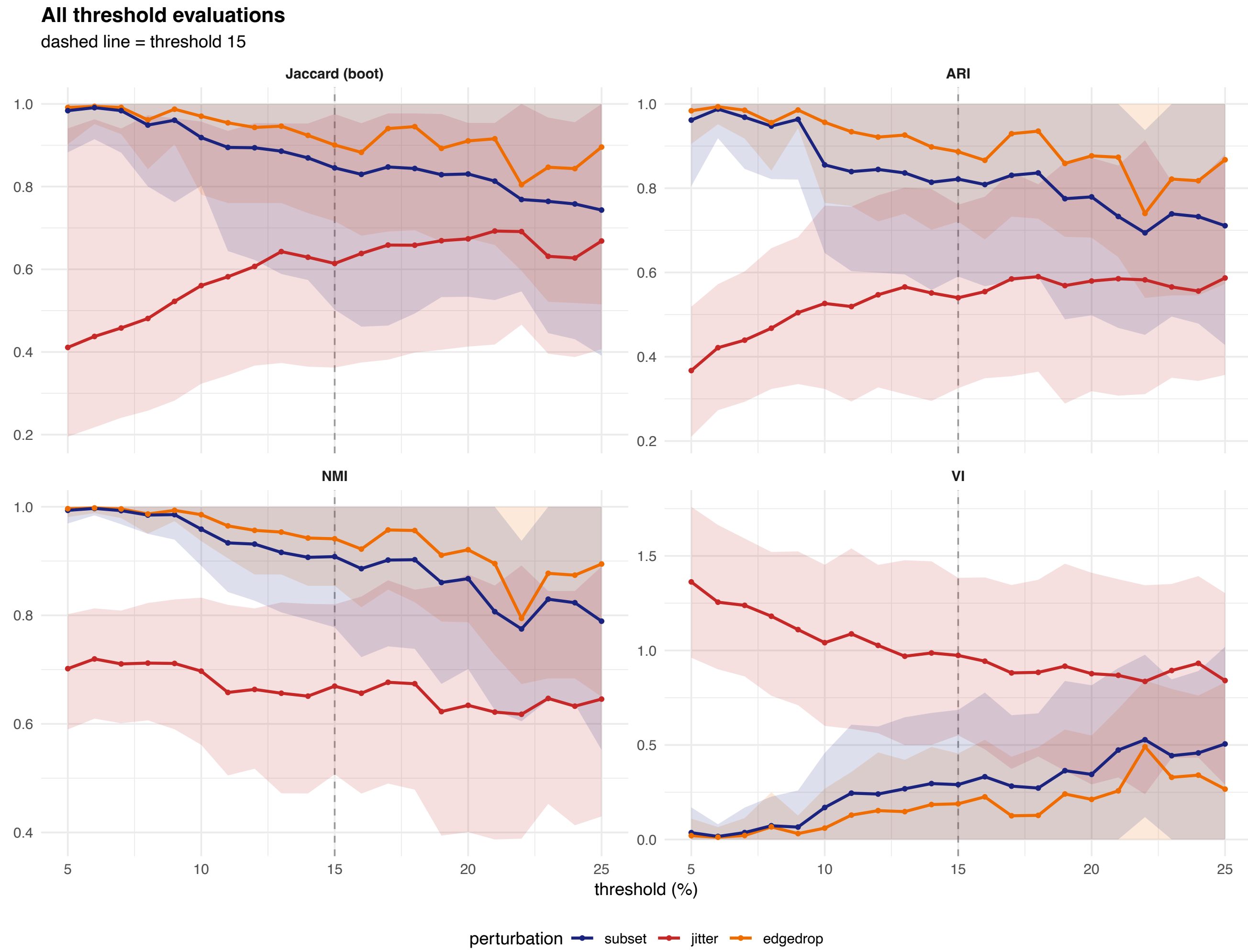

Supplementary Figure 8. Threshold stability across candidate clustering thresholds. Cluster stability was evaluated across graph thresholds retaining the top 5% to 25% of correlations. For each threshold, stability was assessed using 500 bootstrap iterations under three perturbation procedures: subset resampling, edge drop, and jitter. Stability was quantified using Jaccard similarity, adjusted Rand index (ARI), normalized mutual information (NMI), and variation of information (VI). Lines show mean stability estimates and shaded bands indicate confidence intervals across bootstrap iterations. The dashed vertical line denotes the selected 15% threshold. Higher values indicate greater stability for Jaccard, ARI, and NMI, whereas lower values indicate greater stability for VI. Overall, stability estimates showed overlapping confidence intervals across most thresholds, supporting robust clustering stability across a range of plausible threshold choices.

Terms	th5	th6	th7	th8	th9	th10	th11	th12	th13	th14	th15	th16	th17	th18	th19	th20	th21	th22	th23	th24	th25
affect	C1	C1	C1	C1	C1	C1	C1	C1	C1	C1	C1	C1	C1	C1	C1	C1	C1	C1	C1	C1	C1
affective	C1	C1	C1	C1	C1	C1	C1	C1	C1	C1	C1	C1	C1	C1	C1	C1	C1	C1	C1	C1	C1
anticipation	C3	C3	C3	C3	C3	C3	C3	C3	C3	C3	C3	C3	C3	C3	C3	C3	C3	C3	C3	C3	C3
anxiety	C1	C1	C1	C1	C1	C1	C1	C1	C1	C1	C1	C1	C1	C1	C1	C1	C1	C1	C1	C1	C1
arousal	C1	C1	C1	C1	C1	C1	C1	C1	C1	C1	C1	C1	C1	C1	C1	C1	C1	C1	C1	C1	C1
attention	C4	C4	C4	C4	C4	C2	C2	C2	C2	C2	C4	C4	C4	C4	C4	C4	C4	C4	C4	C4	C4
aversive	C1	C1	C1	C1	C1	C1	C1	C1	C1	C1	C1	C1	C1	C1	C3	C3	C3	C1	C3	C3	C3
cognition	C4	C4	C4	C4	C4	C4	C1	C1	C1	C1	C1	C1	C1	C1	C1	C1	C1	C1	C1	C1	C4
cognitive	C4	C4	C4	C4	C4	C2	C2	C2	C2	C2	C4	C4	C4	C4	C4	C4	C4	C4	C4	C4	C4
cognitiveControl	C4	C4	C4	C4	C4	C2	C2	C2	C2	C2	C4	C4	C4	C4	C4	C4	C4	C4	C4	C4	C4
cognitiveImpairment	C4	C4	C4	C4	C4	C4	C4	C4	C4	C4	C5	C2	C2	C2	C4	C4	C4	C4	C2	C2	C2
contextual	C4	C4	C4	C4	C4	C4	C4	C4	C4	C4	C5	C5	C5	C5	C2	C2	C4	C4	C2	C2	C2
control	C4	C4	C4	C4	C4	C4	C4	C4	C4	C4	C5	C4	C4	C4	C4	C4	C4	C4	C4	C4	C4
decision	C4	C3	C3	C3	C3	C3	C3	C3	C3	C3	C3	C3	C3	C3	C3	C3	C3	C3	C3	C3	C3
depression	C4	C4	C4	C4	C4	C4	C1	C1	C1	C1	C1	C1	C1	C1	C1	C1	C1	C1	C1	C1	C1
depressiveDisorder	C4	C4	C4	C4	C4	C4	C4	C4	C4	C4	C1	C1	C1	C1	C1	C1	C1	C1	C1	C1	C1
disorder	C4	C4	C4	C4	C4	C4	C4	C4	C1	C1	C1	C1	C1	C1	C1	C1	C1	C1	C1	C1	C1
emotional	C1	C1	C1	C1	C1	C1	C1	C1	C1	C1	C1	C1	C1	C1	C1	C1	C1	C1	C1	C1	C1
encoding	C2	C2	C2	C2	C2	C2	C2	C2	C2	C2	C2	C2	C2	C2	C4	C4	C4	C4	C2	C2	C2
episodicMemory	C2	C2	C2	C2	C2	C2	C2	C2	C2	C2	C2	C2	C2	C2	C4	C4	C4	C4	C2	C2	C2
executive	C4	C4	C4	C4	C4	C2	C2	C2	C2	C2	C4	C4	C4	C4	C4	C4	C4	C4	C4	C4	C4
executiveControl	C4	C4	C4	C4	C4	C4	C4	C4	C2	C2	C4	C4	C4	C4	C4	C4	C4	C4	C4	C4	C4
fear	C1	C1	C1	C1	C1	C1	C1	C1	C1	C1	C1	C1	C1	C1	C1	C1	C1	C1	C1	C1	C1
inhibition	C4	C4	C4	C4	C4	C4	C4	C4	C4	C4	C5	C5	C5	C5	C2	C2	C2	C2	C5	C5	C4
interference	C4	C4	C4	C4	C4	C2	C2	C2	C2	C2	C4	C4	C4	C4	C4	C4	C4	C4	C4	C4	C4
learning	C4	C4	C3	C3	C3	C3	C3	C3	C3	C3	C3	C3	C3	C3	C3	C3	C3	C4	C3	C3	C3
loss	C3	C3	C3	C3	C3	C3	C3	C3	C3	C3	C3	C3	C3	C3	C3	C3	C3	C3	C3	C3	C3
memories	C2	C2	C2	C2	C2	C2	C2	C2	C2	C2	C2	C2	C2	C2	C4	C4	C1	C4	C2	C2	C2
memory	C2	C2	C2	C2	C2	C2	C2	C2	C2	C2	C4	C4	C4	C4	C4	C4	C4	C4	C4	C4	C4
memoryPerformance	C4	C4	C4	C2	C2	C2	C2	C2	C2	C2	C2	C2	C2	C2	C4	C4	C4	C4	C2	C2	C2
memoryTask	C4	C4	C4	C4	C4	C2	C2	C2	C2	C2	C4	C4	C4	C4	C4	C4	C4	C4	C4	C4	C4
mood	C4	C4	C4	C4	C4	C4	C1	C1	C1	C1	C1	C1	C1	C1	C1	C1	C1	C1	C1	C1	C1
personal	C4	C4	C4	C4	C2	C2	C2	C2	C2	C2	C2	C2	C2	C2	C4	C4	C4	C4	C2	C2	C2
reactivity	C1	C1	C1	C1	C1	C1	C1	C1	C1	C1	C1	C1	C1	C1	C1	C1	C1	C1	C1	C1	C1
regulation	C1	C1	C1	C1	C1	C1	C1	C1	C1	C1	C1	C1	C1	C1	C1	C1	C1	C1	C1	C1	C1
responseInhibition	C4	C4	C4	C4	C4	C4	C4	C4	C4	C4	C5	C5	C5	C5	C2	C2	C2	C2	C5	C5	C4
retrieval	C2	C2	C2	C2	C2	C2	C2	C2	C2	C2	C2	C2	C2	C2	C4	C4	C4	C4	C4	C4	C4
reward	C3	C3	C3	C3	C3	C3	C3	C3	C3	C3	C3	C3	C3	C3	C3	C3	C3	C1	C3	C3	C3
social	C4	C4	C1	C1	C1	C1	C1	C1	C1	C1	C1	C1	C1	C1	C1	C1	C1	C1	C1	C1	C1
stress	C4	C4	C4	C4	C4	C4	C4	C1	C1	C1	C1	C1	C1	C1	C1	C1	C1	C1	C1	C1	C1
workingMemory	C2	C2	C2	C4	C4	C2	C2	C2	C2	C2	C4	C4	C4	C4	C4	C4	C4	C4	C4	C4	C4

Supplementary Figure 9. Cluster assignment stability across candidate graph thresholds. Terms are shown in rows, and candidate graph thresholds retaining the top 5% to 25% of correlations are shown in columns. At each threshold, communities were identified using the fast-greedy clustering algorithm. Cell labels indicate the assigned cluster, with "C" denoting cluster and the accompanying number denoting the cluster identity. Cell colors correspond to cluster assignment and are used to visualize consistency or reassignment of terms across thresholds. Overall, many terms retained stable cluster assignments across the tested threshold range, whereas a subset of terms showed threshold-dependent reassignment, particularly near transitions between adjacent clustering solutions.

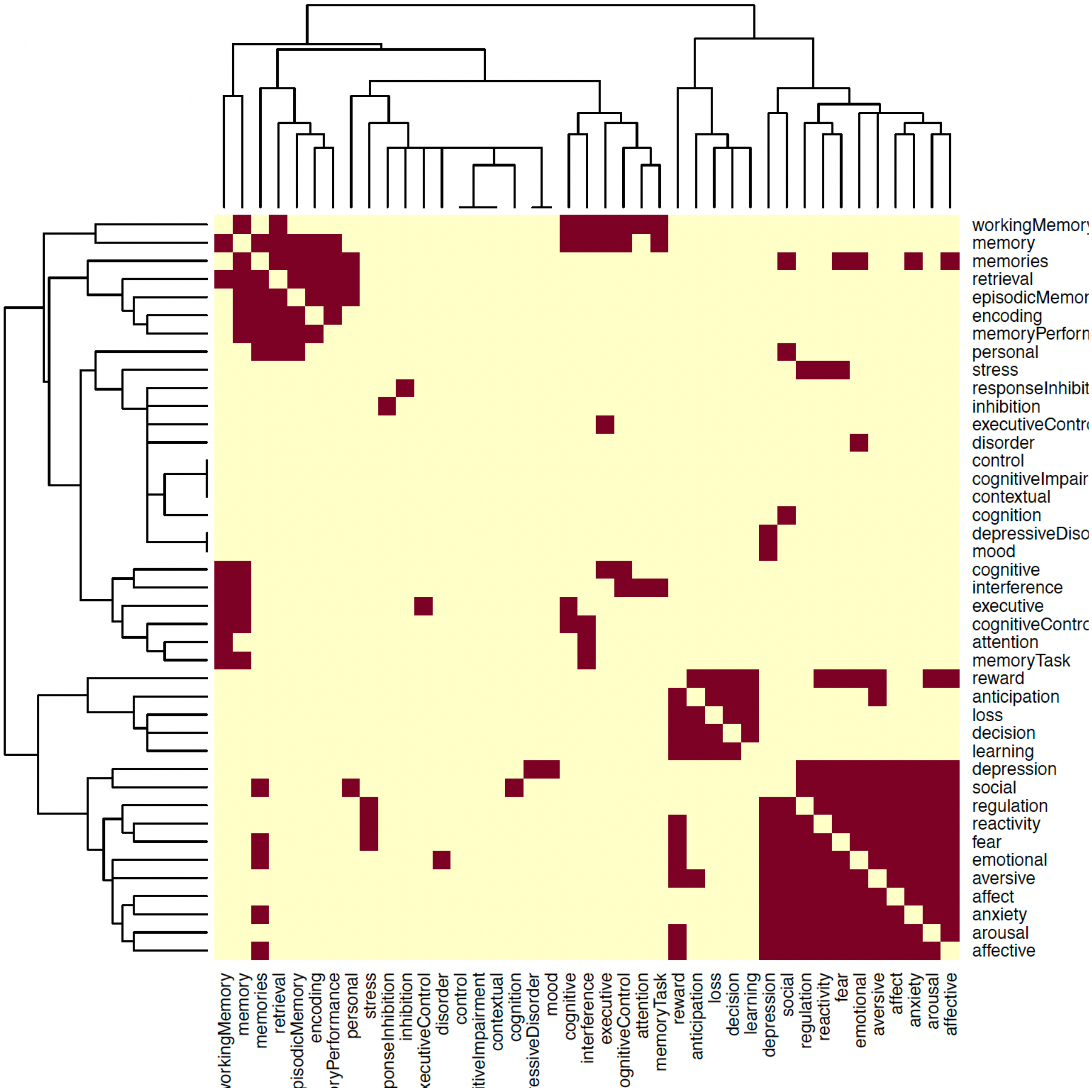

Supplementary Figure 10. Binarized heatmap of fast greedy community detection. Plot shows the unique correlations between the NeuroSynth brain maps were examined and ranked, thresholded at the top 15% of correlations, and retained if above the threshold.

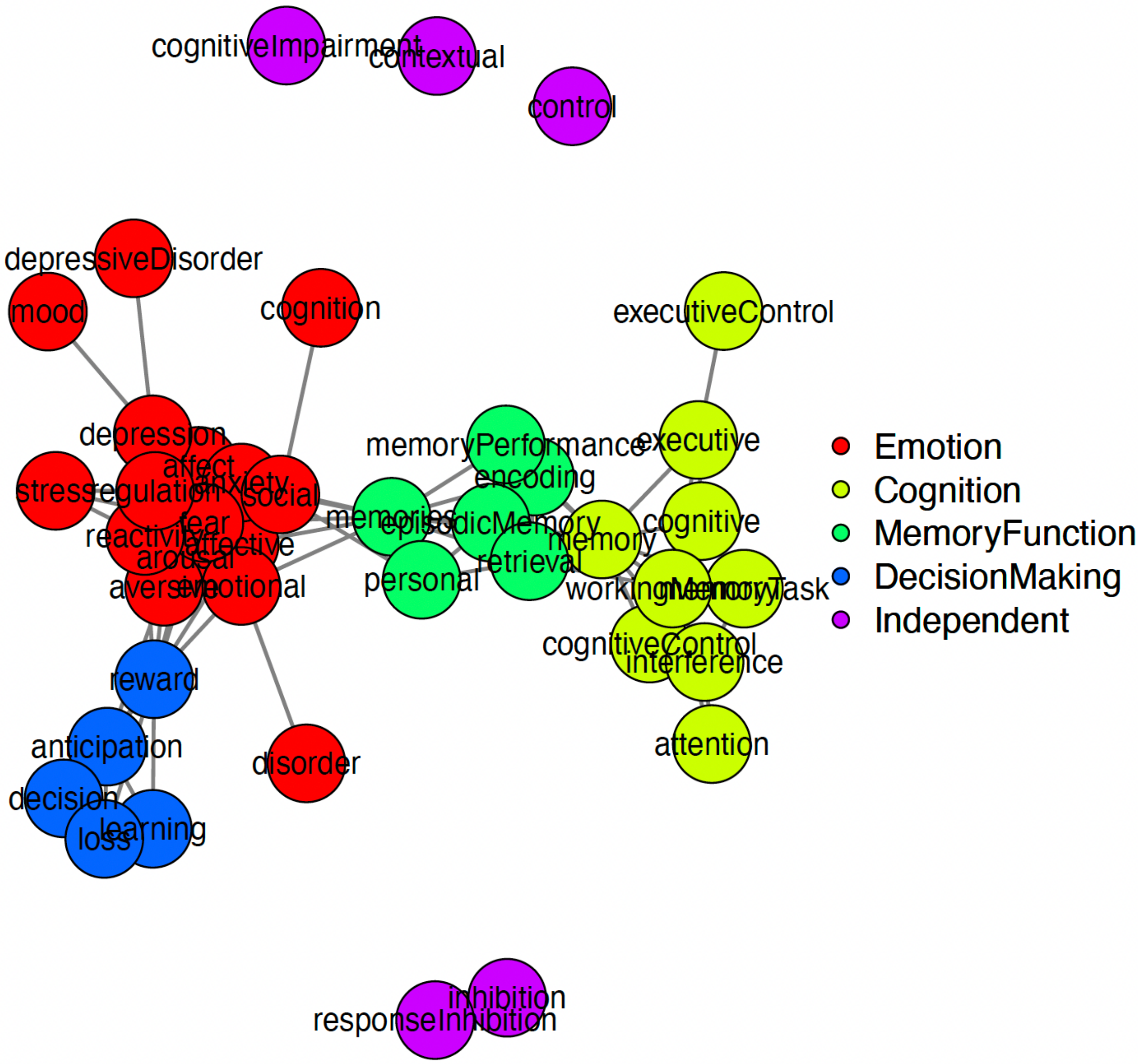

Supplementary Figure 11. Graph network representing the binarized (top 15%) connections. Clustered (fast greedy) concepts are denoted in colored circles.

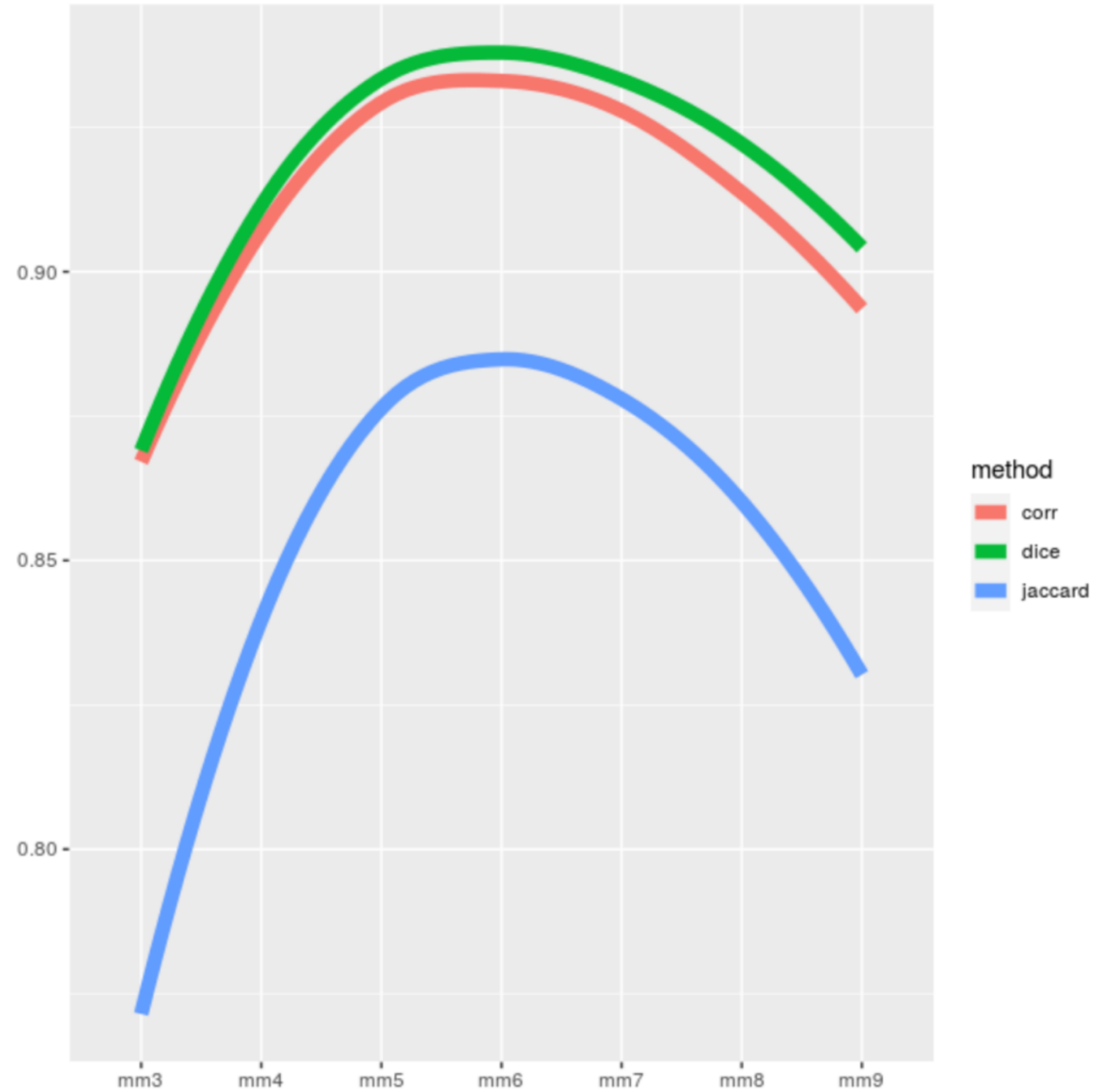

Supplementary Figure 12. Evaluation of ROI radius for constructing PTSD study-specific overlap maps. Spherical ROI radii ranging from 3 to 9 mm were evaluated to determine the radius that produced the most representative overlap between PTSD study-specific brain maps and the Concept Brain Network maps. For each radius, mean similarity was examined using correlation, Dice coefficient, and Jaccard index. Across metrics, similarity increased from smaller radii and peaked around 6 mm before declining at larger radii, indicating that a 6 mm radius provided the strongest overall correspondence while avoiding overly broad spatial overlap. Based on these results, and because 6 mm is the radius used in Neurosynth coordinate-based map generation, 6 mm spherical ROIs were selected for the primary PTSD study map analyses.
